# Effect of Darapladib Treatment on Endarterectomy Carotid Plaque Lipoprotein-Associated Phospholipase A_2_ Activity: A Randomized, Controlled Trial

**DOI:** 10.1371/journal.pone.0089034

**Published:** 2014-02-20

**Authors:** Joel L. Johnson, Yi Shi, Rose Snipes, Salim Janmohamed, Timothy E. Rolfe, Bill Davis, Anthony Postle, Colin H. Macphee

**Affiliations:** 1 GlaxoSmithKline, Research Triangle Park, North Carolina, United States of America; 2 Thomas Jefferson University, Philadelphia, Pennsylvania, United States of America; 3 GlaxoSmithKline, Stockley Park, Middlesex, United Kingdom; 4 GlaxoSmithKline, Addenbrook, Cambridge, United Kingdom; 5 University of Southampton, Southampton, United Kingdom; 6 GlaxoSmithKline, King of Prussia, Pennsylvania, United States of America; Indiana University Richard M. Fairbanks School of Public Health, United States of America

## Abstract

**Background:**

The aim of this study was to assess the effects of darapladib, a selective oral investigational lipoprotein-associated phospholipase A_2_ inhibitor, on both plasma and plaque lipoprotein-associated phospholipase A_2_ activity.

**Methods:**

Patients undergoing elective carotid endarterectomy were randomized to darapladib 40 mg (n = 34), 80 mg (n = 34), or placebo (n = 34) for 14 days, followed by carotid endarterectomy 24 hours after the last dose of study medication.

**Results:**

Darapladib 40 mg and 80 mg reduced plasma lipoprotein-associated phospholipase A_2_ activity by 52% and 81%, respectively, versus placebo (both *P*<0.001). Significant reductions in plaque lipoprotein-associated phospholipase A_2_ activity were also observed compared with placebo (*P*<0.0001), which equated to a 52% and 80% decrease compared with placebo. No significant differences were observed between groups in plaque lysophosphatidylcholine content or other biomarkers, although a dose-dependent decrease in plaque matrix metalloproteinase-9 mRNA expression was observed with darapladib 80 mg (*P* = 0.053 vs placebo). In a post-hoc analysis, plaque caspase-3 (*P*<0.001) and caspase-8 (*P*<0.05) activity were found to be significantly lower in the darapladib 80-mg group versus placebo. No major safety concerns were identified in the study.

**Conclusions:**

Short-term treatment (14±4 days) with darapladib produced a robust, dose-dependent reduction in plasma lipoprotein-associated phospholipase A_2_ activity. More importantly, darapladib demonstrated placebo-corrected reductions in carotid plaque lipoprotein-associated phospholipase A_2_ activity of similar magnitude. Darapladib was generally well tolerated and no safety concerns were identified. Additional studies of longer duration are needed to explore whether these pharmacodynamic effects are associated with improved clinical outcomes, as might be hypothesized.

**Trial Registration Information:**

Name of Registry 1: ClinicalTrials.gov

Registry Number 1: NCT01916720

Trial URL in Registry Database 1: www.clinicaltrials.gov/ct2/show/NCT01916720

Name of Registry 2: GSK Clinical Study Register

Registry Number 2∶480848/010

Trial URL in Registry Database 2: www.gsk-clinicalstudyregister.com/result_detail.jsp?protocolId=480848%2F010&studyId=74F5DB65-4661-4FA8-91D4-EBF78D769F24&compound=darapladib&type=Compound&letterrange=A-F

## Introduction

Despite medical and scientific advances over the past 15 years, the burden associated with atherosclerotic cardiovascular (CV) disease remains unacceptably high. Broadening treatment targets to include lipid-related risk factors beyond currently recognized CV risk markers may help further improve clinical outcomes [1]. Additional processes within the vessel wall, particularly inflammatory responses, contribute to plaque destabilization, atherothrombosis, and the clinical sequelae. Mediators of these processes therefore represent potential novel treatment targets for reducing CV risk and the burden associated with atherosclerotic CV disease.

Lipoprotein-associated phospholipase A_2_ (Lp-PLA_2_) is an emerging CV risk marker that may play an important pathogenetic role in mediating inflammatory processes that contribute to plaque vulnerability and rupture [2]. Although Lp-PLA_2_ has been described as a platelet-activating factor (PAF) acetylhydrolase [3], accumulating evidence suggests that the products of Lp-PLA_2_, lysophosphatidylcholine (lysoPC) and oxidized nonesterified fatty acids, can elicit a broad range of proinflammatory and pro-apoptotic effects [4]. Lp-PLA_2_ is produced and secreted by activated macrophages and other inflammatory cells implicated as playing a central role in atherothrombosis [5]. More recently, it has been shown that the so-called proinflammatory macrophage (M1 phenotype) expresses and secretes greater quantities of Lp-PLA_2_ when compared with M2 polarized macrophages [6]. Since M1 inflammatory macrophages tend to be located within the lipid core of vulnerable plaques [7], the association described above presumably explains why Lp-PLA_2_ is highly upregulated in macrophages located within the necrotic core and fibrous cap of vulnerable and ruptured human coronary plaques, but not within stable lesions [8]. Indeed, in patients who underwent carotid endarterectomy, levels of Lp-PLA_2_ and one of its products, lysoPC, were higher in plaques from patients who had experienced CV events compared with those who had not, suggesting that Lp-PLA_2_ was a key component of a causal pathway for plaque vulnerability [9]–[11]. A recent analysis of carotid plaque Lp-PLA_2_ and lysoPC content demonstrated a highly statistically significant correlation of both biomarkers with various plaque M1 macrophage-related proinflammatory cytokines such as IL-6, tumor necrosis factor-α and monocyte chemoattractant protein-1 [11]. Finally, a pro-atherothrombotic role for Lp-PLA_2_ is further supported by a body of epidemiologic evidence suggesting a greater risk of CV events with elevated plasma Lp-PLA_2_ [12].

Darapladib is an oral, investigational, highly potent, and selective Lp-PLA_2_ inhibitor that has been demonstrated to reduce atherosclerosis in a diabetic and hypercholesterolemic porcine model of accelerated coronary atherosclerosis [13] as well as in ApoE-deficient mice [14]. Administration of darapladib in the pig model not only inhibited coronary artery lesion development, but more profoundly reduced progression to advanced coronary lesions. A similar observation was noted in the IBIS-2 (Integrated Biomarker and Imaging Study 2) clinical trial, which compared the effects of 12 months of darapladib treatment with placebo on plasma C-reactive protein levels and coronary atheroma composition and deformability in 330 patients with angiographically documented coronary artery disease [15]. Although the primary end points of the study were not met, and darapladib did not affect total atheroma volume, treatment did halt the increase in necrotic core volume as assessed by intravascular ultrasound virtual histology, suggesting a stabilization of the overall plaque. Whether these changes will translate into differences in clinical outcomes awaits the results of two large, fully enrolled, ongoing phase III trials: STABILITY (Stabilization of Atherosclerotic Plaque by Initiation of Darapladib Therapy Trial, NCT00799903), involving 15,828 patients with coronary heart disease [16], and SOLID-TIMI 52 (Stabilization of Plaques Using Darapladib - Thrombolysis in Myocardial Infarction 52 Trial, NCT01000727), involving 13,026 subjects with a recent history of acute coronary syndrome [17].

The primary objective of the current study was to test the hypothesis that darapladib could reduce the elevated Lp-PLA_2_ activity within advanced atherosclerotic (carotid) plaques in a dose-dependent manner. The secondary objectives were more exploratory in nature, given the very short treatment period, and focused on changes of several potentially relevant plaque biomarkers, including an analysis of species of lysoPC. A portion of the data has been presented in abstract format [18].

## Methods

The protocol for this trial and supporting CONSORT checklist are available as supporting information; see Protocol S1 and CONSORT Checklist S1.

### Patients

Men and women 35 years or older with carotid atherosclerosis requiring endarterectomy were eligible for participation in this study. Major exclusion criteria were a history of myocardial infarction within 4 weeks prior to screening, initiation or alteration of a lipid-modifying regimen within 4 weeks prior to randomization, and a history of unstable angina without evolution of myocardial infarction (based on electrocardiogram or clinical laboratory marker criteria), but with transient ST elevation or depression, or T-wave inversion associated with pain; or in the absence of electrocardiogram changes, a documented history of prior myocardial infarction or angiographic coronary artery stenosis. Patients were recruited from January 27, 2003, until June 11, 2003, and followed up until July 14, 2003.

### Study Design and Conduct

This randomized, double-blind, placebo-controlled, parallel-group study was conducted in 20 participating study centers throughout Europe (ClinicalTrials.gov identifier: NCT01916720; www.clinicaltrials.gov/ct2/show/NCT01916720; GSK trial identifier: 480848/010; www.gsk-clinicalstudyregister.com/result_detail.jsp?protocolId=480848%2F010&studyId=74F5DB65-4661-4FA8-91D4-EBF78D769F24&compound=darapladib&type=Compound&letterrange=A-F). Eligible subjects were randomly assigned (1∶1∶1 fashion) to treatment with either darapladib 40 mg or 80 mg or matching placebo once daily. The randomization was stratified by statin use and gender. Allocation of eligible subjects was determined using a computer-generated list of random numbers. A central randomization schedule was created by the GSK study statistician using CMS, a GlaxoSmithKline internal randomization system, with a 1∶1∶1 allocation ratio between 3 treatment groups, using the block size of 4. The GSK Registration and Medication Ordering System (RAMOS) was used to sequentially allocate treatments to subjects as per the randomization generated within CMS. RAMOS also provided central allocation of drug supplies. Study medication was dispensed according to the protocol to randomized subjects under the supervision of the investigator: placebo patients received 2 bottles of placebo (pbo) and took two tablets from each bottle (i.e., 4×pbo); 40 mg patients received 1 bottle of 20 mg tablets and 1 bottle of placebo and took two tablets from each bottle (i.e., 2×20 mg+2×pbo); 80 mg patients received 2 bottles of 20 mg and took two tablets from each bottle (i.e., 4×20 mg). Subjects, physicians, and site staff associated with the study, as well as GlaxoSmithKline teams, were blinded and unaware of the allocated treatment.

The duration of treatment was 14±4 days and was planned to immediately precede elective carotid endarterectomy. The last dose of study medication was administered on the day prior to the surgical procedure, which was carried out according to the guidelines set forth by the local institution. Data were collected by the investigators at each site and analyzed by the sponsor.

### Ethics Statement

Subjects provided written, informed consent, and the protocol was approved by each study site’s respective ethics committee: Ethical Committee of the Hospital District of Helsinki and Uusimaa, ophthalmology, otorhinolaryngology, neurology and neurosurgery, Helsinki, Finland; CCPPRB Paris-Pitiè-Salpétrière, Hôpital Pité-Salpétrière, Pavillon Jacquart, Paris, France; Universitätsklinikum Benjamin-Franklin Ethikkommission, Berlin, Germany; Otto-von Guericke-Universität Ethikkommission, Magdeburg, Germany; Ethikkommission der Westfälischen Wilhelms-Universität Münster, Münster, Germany; Landesärztekammer Hessen Ethikkommission, Frankfurt, Germany; Medisch Ethische Toetsingscommissie UMC Utrecht, Utrecht, Netherlands; Sint Antonius Ziekenhuis TME, Nieuwegein, Netherlands; Komisja Bioetyczna CMKP, Warsaw, Poland; Comité Etico de Investigación Clínica, H. Clínico de Barcelona, Barcelona, Spain; SERGAS, Comité Etico de Investigación Clínica, Santiago de Compostela, Spain; Comité Etico de Investigación Clínica, Servicio Farmacología Clínica, H. San Creu y San Pau, Barcelona, Spain; Comité Etico de Investigación Clínica, H de Bellvitge, Barcelona, Spain; Comité Etico de Investigación Clínica, H Central de Asturias, Oviedo, Spain; Comité Etico de Investigación Clínica, H. Vall d.Hebrón, Barcelona, Spain; United Bristol Healthcare Trust Ethics Committee, UBHT Headquarters, Bristol, UK; East Dorset Local Research Ethics Committee, Poole Hospital NHS Trust, Poole, UK; Bath Local Research Ethics Committee, Royal United Hospital, Bath, UK. This study was conducted in accordance with Good Clinical Practice Guidelines and guiding principles of the Declaration of Helsinki.

### Measurements

The excised plaque was rinsed in saline, frozen in liquid nitrogen, transferred to a 50 mL Falcon tube, and stored at −70°C or in liquid nitrogen. Specimens were shipped using dry ice transport to Quest Diagnostics Clinical Trials (Heston, Middlesex, United Kingdom) for storage. Plaque samples were shipped to GlaxoSmithKline for analysis of Lp-PLA_2_ activity (Human Biomarker Centre, Ware, Hertfordshire, United Kingdom), phospholipids (Human Biomarker Centre/University of Southampton), and biomarker gene expression (CD68, CD3, α-actin, CD40 ligand [CD40L], matrix metalloproteinase [MMP]-2, MMP-9, plasminogen activator inhibitor [PAI]-1, intercellular adhesion molecule [ICAM]-1, interleukin [IL]-6, and Lp-PLA_2_; Quantitative Expression and Genomic Histology Department, Stevenage, Hertfordshire, United Kingdom). Lp-PLA_2_ activity and lysoPCs were assessed in excised lesions as previously described [13] and included lysoPC 16∶1, lysoPC 16∶0, lysoPC 18∶2, lysoPC 18∶1, lysoPC 18∶0, lysoPC 20∶4, lysoPC 20∶3, and lysoPC 22∶6. The expression of biomarkers was captured as the cDNA copy numbers from TaqMan™ (Applied Biosystems, Inc., Foster City, California, USA) real-time polymerase chain reaction analyses.

Blood samples were obtained immediately prior to the first dose (i.e., baseline) and again after 14±4 days of treatment with study medication. Blood samples were centrifuged at 4°C, frozen at -70°C, and shipped to Quest Diagnostics Clinical Trials (using dry ice transport) for storage. Clinical laboratory chemistries and plasma biomarker protein concentrations (high-sensitivity C-reactive protein, CD40L, sICAM-1, E-selectin, PAI-1, and MMP-9) were assessed at Quest, whereas frozen plasma samples were shipped to GlaxoSmithKline (Human Biomarker Center, Philadelphia, Pennsylvania, USA) for analysis of plasma Lp-PLA_2_ activity. Lp-PLA_2_ activity in plaque and plasma samples was measured using a radiometric assay described previously, with [^3^H]-PAF as a substrate [19] and normalized to protein content for plaque measurements.

Based on findings suggesting that products of Lp-PLA_2_ activity could promote macrophage apoptosis [20], a post-hoc analysis was performed using stored plaque specimens to assess the effect of treatment with darapladib on markers of apoptosis, plaque caspase activity (effector/executioner caspase-3 and initiator caspase-8). Stored specimens were available only from a subset of patients from the study (for caspase-3, n = 23, n = 21, and n = 15 for the placebo, 40-mg, and 80-mg groups, respectively; for caspase-8, n = 10, n = 17, and n = 10, respectively). Colorimetric assays (BioVision, Mountain View, California, USA) were used to measure caspase-3 and caspase-8 activity, with DEVD-pNA and IETD-pNA as the substrates, respectively, and the values are presented as geometric mean (±SEM) activity units per milligram of protein (AU/mg).

Safety assessments included clinical laboratory chemistries on blood samples obtained at baseline, on Day 7 (±2), on Day 15, and 28 (±7) days following the last dose of study medication. Physical examinations (during which vital signs were obtained) were performed at these same points during the trial. An electrocardiogram was obtained at baseline, on Day 15, and 28 (±7) days following the last dose of study medication.

### Statistical Analyses

The primary end point measure of the study was Lp-PLA_2_ activity within the atherosclerotic plaque removed during carotid endarterectomy following 14 (±4) days of treatment with darapladib, adjusted by sex and statin use. Sample size calculations indicated that 33 enrolled subjects would be needed for each treatment group to achieve 27 evaluable subjects to detect a 50% difference in plaque Lp-PLA_2_ activity between darapladib (either dose) and placebo (with a standard deviation of 0.7 on a log-transformed scale), with 90% power. Secondary efficacy end points included the percent inhibition of Lp-PLA_2_ activity in blood, and the concentration and/or mRNA expression of other biomarkers.

A Bonferroni adjustment was used for primary efficacy analyses to allow for a comparison of each darapladib dose to placebo, with a 2.5% significance level for each comparison with placebo (maintaining an overall 5% significance level for the primary end point). The log-transformed Lp-PLA_2_ activity was analyzed using parametric analysis of covariance (including terms for sex and statin use), and the differences between treatment groups versus placebo in the primary efficacy end point were presented as mean values with 97.5% confidence intervals (CI).

## Results

**Figure 1:**
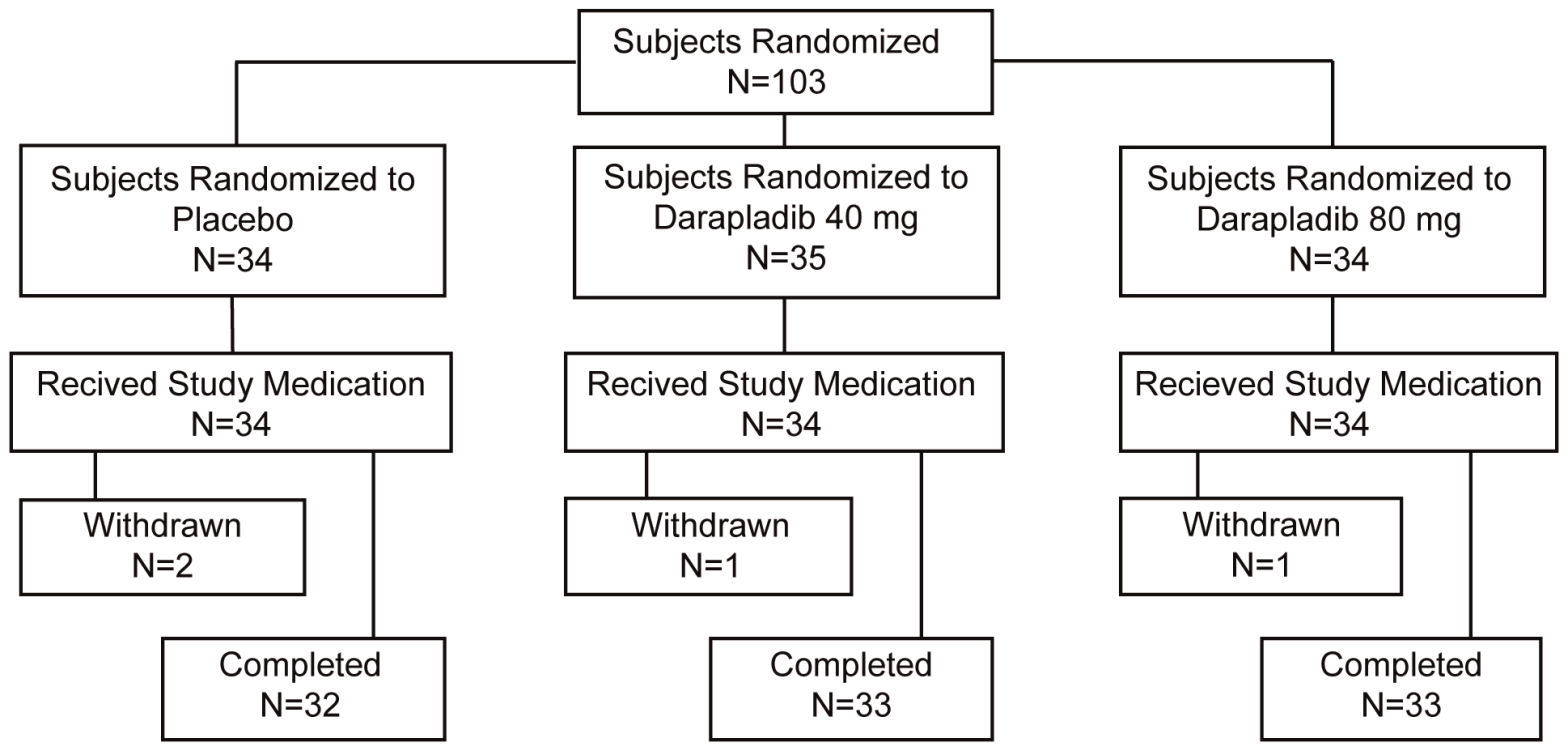
Disposition of patients throughout the carotid endarterectomy study.

One hundred and three eligible subjects were randomly assigned to a treatment group; the disposition of these participants is summarized in Figure 1. One subject assigned to darapladib 40 mg did not receive any study medication because of a protocol deviation (history of asthma). Safety assessments were performed on the remaining 102 subjects (n = 34 per treatment group) who received at least 1 dose of study medication. Overall, 98 (96.1%) subjects completed the study. One subject in the darapladib 80-mg group withdrew from the study prematurely because of an adverse event; this subject experienced a hypertensive crisis while receiving anesthesia in preparation for carotid endarterectomy, 1 day following the completion of study medication. One subject in the placebo group withdrew from the study because the carotid endarterectomy was not performed within 1 day of the last dose of study medication. Two subjects (1 in the darapladib 40-mg group and 1 in the placebo group) withdrew because surgery was not performed due to severe coronary artery disease. The primary efficacy analyses were performed on data from 89 of 103 (86.4%) participants (n = 30 in the placebo group, n = 30 in the darapladib 40-mg group, and n = 29 in the darapladib 80-mg group) who received at least 1 dose of study medication and had evaluable Lp-PLA_2_ activity data from their excised plaques.

**Figure 2:**
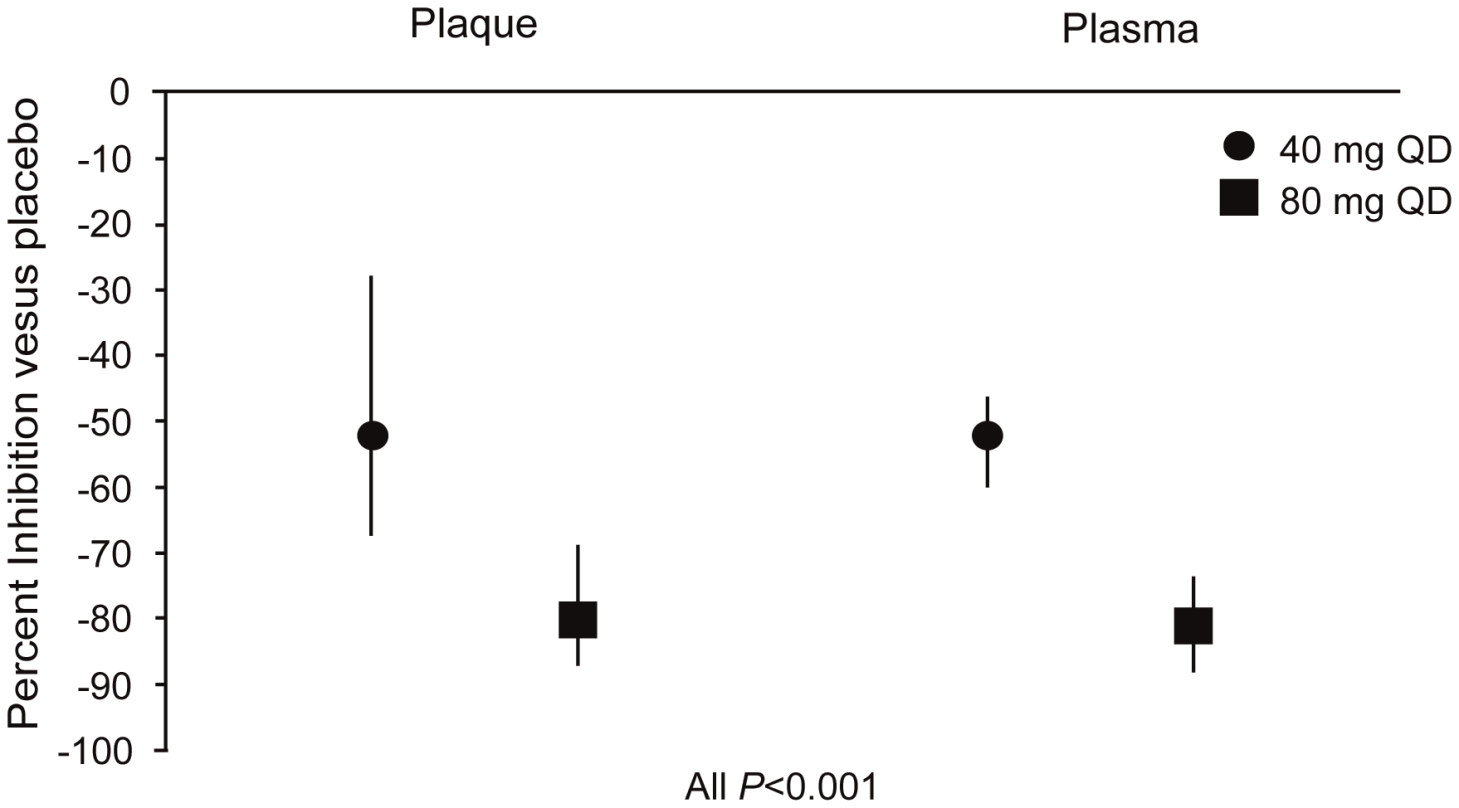
Effect of darapladib treatment (14±4 days) on plaque and plasma lipoprotein-associated phospholipase A_2_ activity. Vertical bars for plaque and plasma data points represent 97.5% and 95% confidence intervals (CI), respectively. Baseline plasma Lp-PLA_2_ activity levels are provided in Table 1. At Day 15, plasma Lp-PLA_2_ activity levels (mean ± SD) for placebo, 40 mg darapladib, and 80 mg darapladib were 141.4±39.3, 63.4±28.3, and 27.0±11.4 nmol/min/ml, respectively. At Day 15, plaque Lp-PLA_2_ activity levels (mean ± SEM) for placebo, 40 mg darapladib, and 80 mg darapladib were 0.94±0.14, 0.26±0.13, and 0.11±0.14 nmol/min/mg protein, respectively.

Demographic characteristics of the study population at baseline are summarized in Table 1. The mean degree of carotid artery stenosis in the study population is consistent with guideline recommendations for endarterectomy. At 24 hours following the last dose of study medication, Lp-PLA_2_ activity in the excised plaque was approximately 52% (97.5% CI, −28% to −68%) lower in the darapladib 40-mg group compared with the placebo group (treatment difference = −0.737 nmol of PAF/min/mg of total protein [97.5% CI, −1.15 nmol/min/mg to −0.32 nmol/min/mg], *P*<0.001). The adjusted mean difference in plaque Lp-PLA_2_ activity between the darapladib 80-mg group and the placebo group was −1.60 nmol of PAF/min/mg of total protein (97.5% CI, −2.02 nmol/min/mg to −1.19 nmol/min/mg, *P*<0.001), which equates to an approximately 80% (97.5% CI, −69% to −87%) reduction in plaque Lp-PLA_2_ activity. The placebo-corrected reduction in plasma Lp-PLA_2_ activity 24 hours following the last dose of study medication was comparable to that observed in plaque (Figure 2). Respectively, treatment with darapladib 40 mg and 80 mg resulted in a 52% (95% CI, −44% to −60%) and an 81% (95% CI, −73% to −89%) reduction in adjusted mean plasma Lp-PLA_2_ activity versus placebo. Consistent with prior studies, there was a significant correlation between baseline plasma Lp-PLA_2_ activity and baseline fasting cholesterol levels (Spearman correlation 0.48, *P*<0.001).

**Table 1:** Summary of demographic characteristics in the carotid endarterectomy study.

	Placebo	Darapladib 40 mg	Darapladib 80 mg
	(N = 34)	(N = 34)	(N = 34)
Age, y (mean ± SD)	66.9±9.0	66.1±7.42	64.3±12.3
Male, n (%)	28 (82.4)	26 (76.5)	25 (73.5)
BMI, kg/m^2^ (mean ± SD)	27.3±3.12	26.6±3.75	26.5±3.58
Caucasian, n (%)	34 (100.0)	34 (100.0)	34 (100.0)
Smoking status, n (%)			
Previous	18 (52.9)	16 (47.1)	19 (55.9)
Current	8 (23.5)	11 (32.4)	7 (20.6)
Never	8 (23.5)	7 (20.6)	8 (23.5)
Diabetic, n (%)	10 (29.4)	11 (32.4)	6 (17.6)
Current cardiovascular conditions, n (%)			
Carotid artery disease	34 (100)	34 (100)	34 (100)
Cerebrovascular disease	34 (100)	34 (100)	34 (100)
Hypertension	20 (58.8)	20 (58.8)	21 (61.8)
Stroke	3 (8.8)	8 (23.5)	9 (26.5)
Coronary artery disease	9 (26.5)	6 (17.6)	3 (8.8)
TIA	5 (14.7)	6 (17.6)	6 (17.6)
Stable angina	7 (20.6)	4 (11.8)	2 (5.9)
Statin use, n (%)	17 (50.0)	17 (50.0)	19 (55.9)
Degree of carotid artery stenosis, % (mean ± SD)	83.8±10.1	83.4±12.2	83.4±10.0
Baseline plasma Lp-PLA_2_ activity*, nmol/min/mL (mean ± SD)	144.1±33.6	133.5±30.9	134.2±30.1
Baseline total cholesterol level, mmol/L (mean ± SD)	5.33±1.10	4.95±0.84	5.30±1.17

BMI = body mass index; TIA = transient ischemic attack.

*Data available from n = 29 subjects in each group.

**Figure 3:**
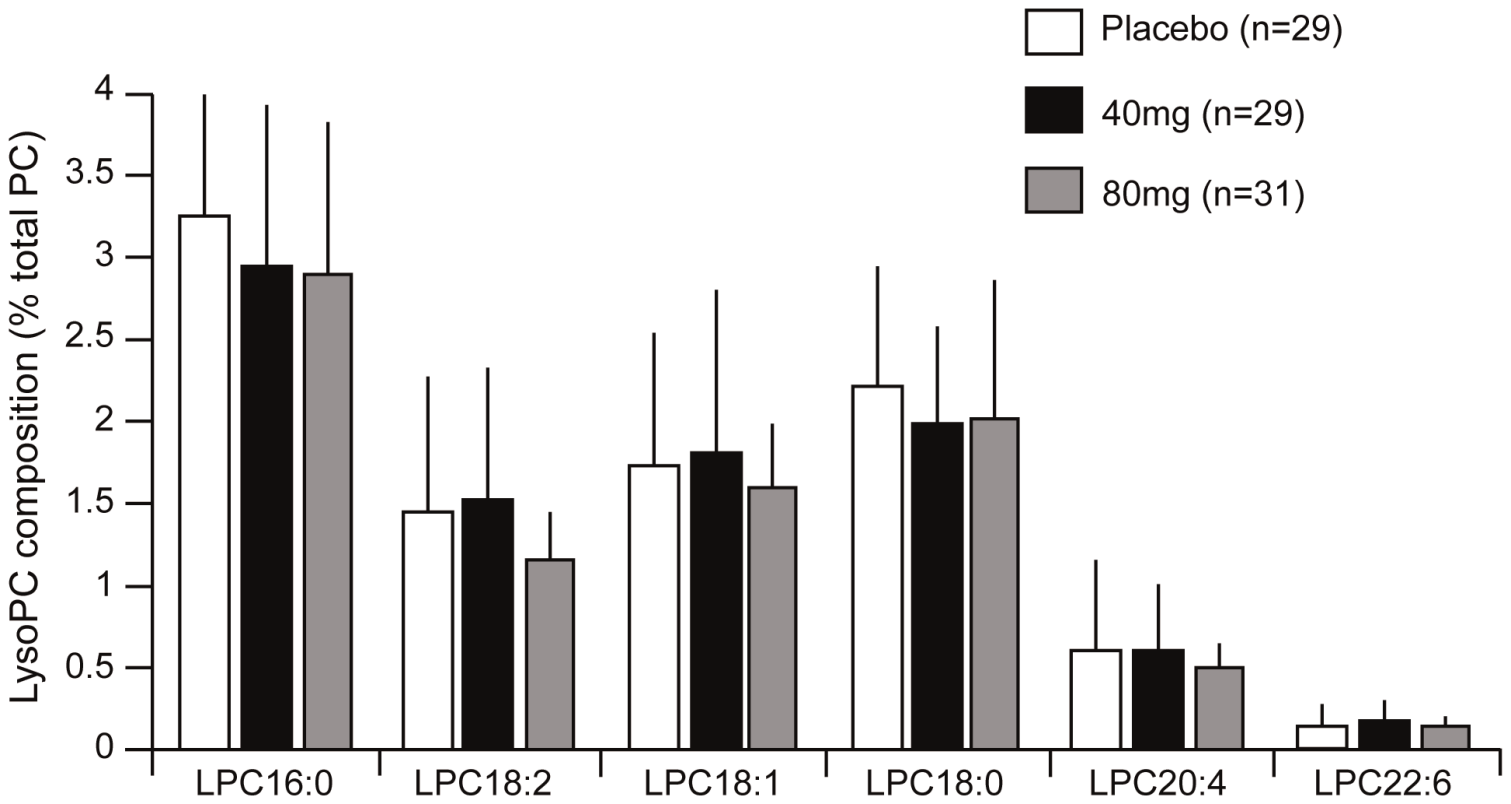
Analysis of plaque lysophosphatidylcholine (lysoPC) content following treatment (14±4 days) with darapladib. Data represent mean ± standard deviation.

No statistically significant differences were observed between treatment groups in the lysoPC content in plaques (Figure 3). The mean plaque concentration of lysoPC (total) at the end of study treatment in the placebo, darapladib 40-mg, and darapladib 80-mg groups was 3.65 (±1.63) µmol/g of tissue, 3.39 (±2.05) µmol/g of tissue, and 3.14 (±1.49) µmol/g of tissue, respectively (*P* = NS). Likewise, no significant differences were observed for the predominant lysoPC species that are known products of Lp-PLA_2_ activity, namely, LPC16∶0, LPC18∶0, and LPC18∶1 (Figure 3) [21].

**Figure 4:**
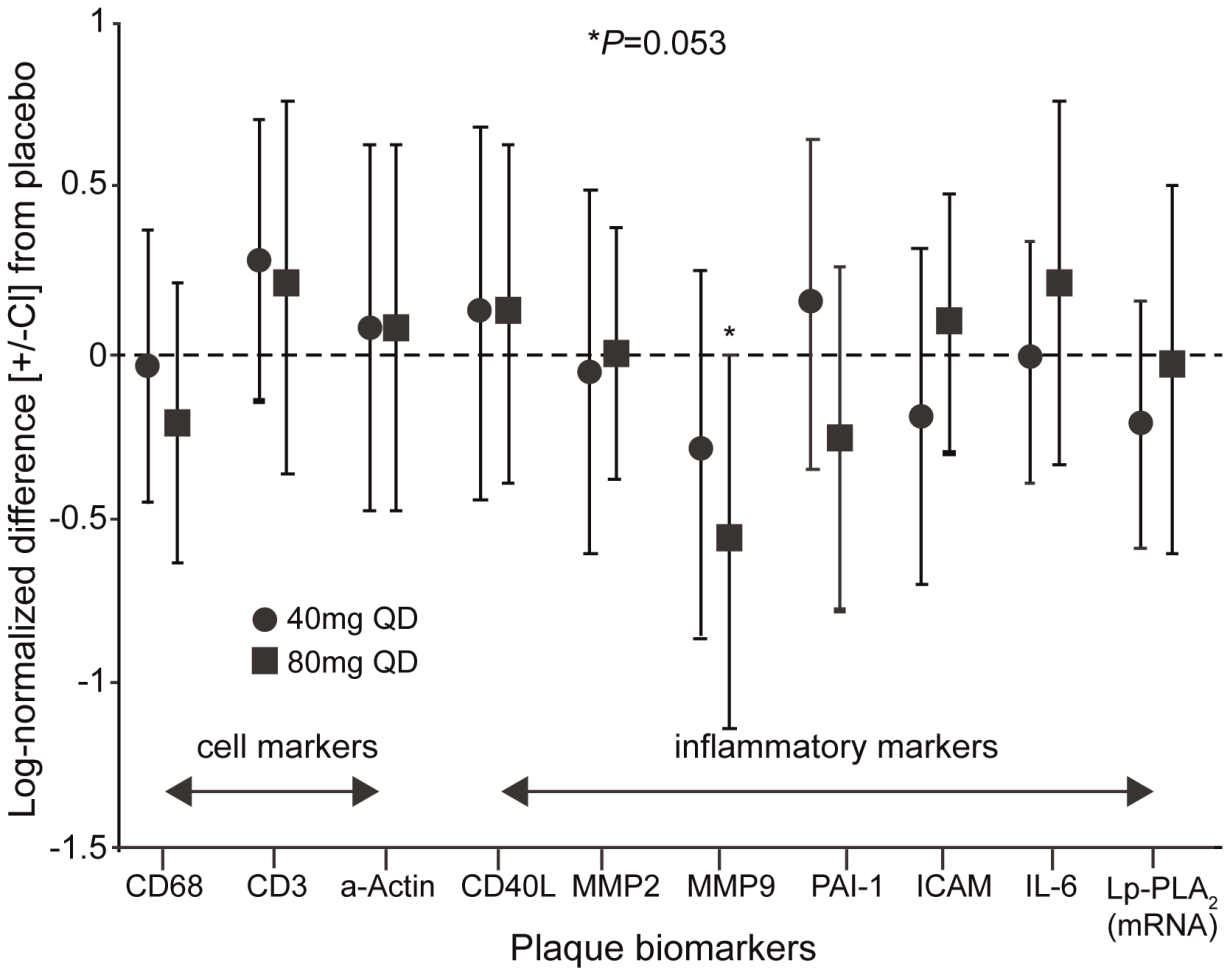
The influence of darapladib treatment (14±4 days) on the expression (mRNA) of several plaque biomarkers. Vertical bars represent 95% confidence intervals (CI). CD40L, CD40 ligand; MMP, matrix metalloproteinase; PAI, plasminogen activator inhibitor; ICAM, intercellular adhesion molecule; IL, interleukin; Lp-PLA, lipoprotein-associated phospholipase A_2_.

No statistically significant differences were observed between the treatment arms for other plasma biomarkers; however, with respect to plaque biomarkers, treatment with darapladib 80 mg produced a ∼33% reduction in the geometric mean plaque MMP-9 mRNA expression compared with placebo that appeared to be dose dependent. The difference between groups was not statistically significant for this (*P* = 0.053 vs placebo, based on absolute differences) or any of the other prespecified biomarkers (Figure 4).

**Figure 5:**
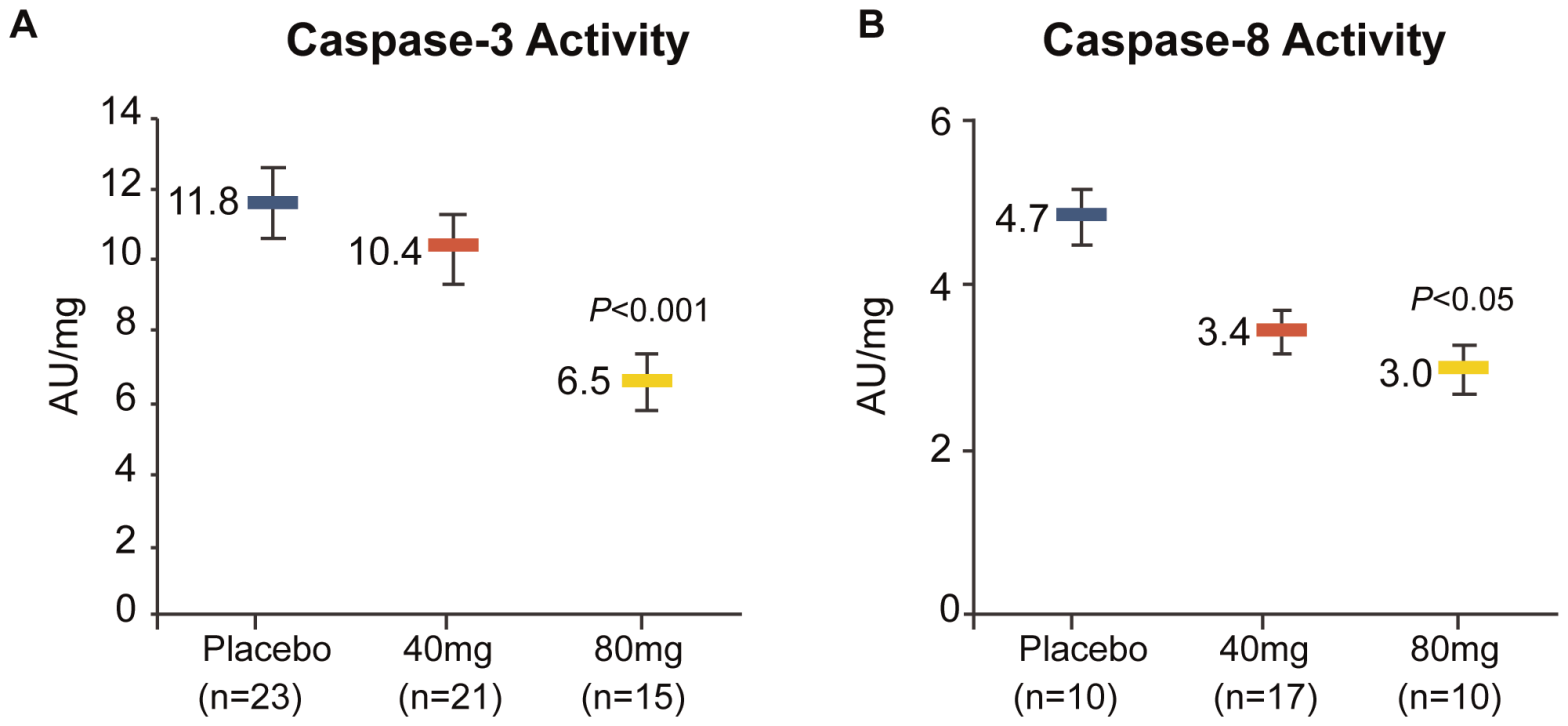
The influence of darapladib treatment (14±4 days) on plaque (A) caspase-3 activity and (B) caspase-8 activity (mean ± SEM). *P* values represent comparisons of darapladib 80 mg with placebo. AU, activity units.

The effects of darapladib on caspase-3 and caspase-8 activity from stored samples are shown in Figure 5. Based on this post-hoc analysis, the activity of both caspases was significantly lower among those receiving darapladib 80 mg compared with placebo (*P*<0.001 for caspase-3 and *P*<0.05 for caspase-8). Using Spearman’s rank method, statistically significant correlations were observed between plasma Lp-PLA_2_ activity and plaque caspase-3 activity (r = 0.51, *P*<0.0001) and between plasma Lp-PLA_2_ activity and plaque caspase-8 activity (r = 0.34, *P* = 0.039).

Treatment with darapladib in this study was generally well tolerated; no clinically meaningful differences were observed between the placebo group and each darapladib group in vital signs, electrocardiograms, or clinical laboratory parameters. The most common adverse events reported during treatment and the post-treatment follow-up period are summarized in Table 2. The most common adverse events with a probable relationship to study medication during the treatment phase were dysgeusia and fecal malodor; however, no subjects withdrew as a result of these events. As mentioned previously, one subject in the darapladib 80-mg group withdrew from the study prematurely because of an adverse event (hypertensive crisis while receiving anesthesia in preparation for carotid endarterectomy); however, this event was considered to be unrelated to the study medication.

**Table 2:** Summary of adverse experiences during treatment (14±4 days) in the carotid endarterectomy study.

	Placebo	Darapladib 40 mg	Darapladib 80 mg
	(N = 34)	(N = 34)	(N = 34)
Deaths, n (%)	0 (0.0)	0 (0.0)	0 (0.0)
Patients with any adverse event, n (%)	5 (14.7)	11 (32.4)	11 (32.4)
Patients with treatment-related adverse event, n (%)	0 (0.0)	3 (8.8)	2 (5.9)
Patients discontinuing treatment from a treatment-related adverse event, n (%)	0 (0.0)	0 (0.0)	0 (0.0)
Patients with serious adverse event, n (%)	0 (0.0)	3 (8.8)	2 (5.9)
Common adverse events, n (%)			
Dysgeusia	0 (0.0)	3 (8.8)	3 (8.8)
Fecal abnormality	0 (0.0)	4 (11.8)	2 (5.9)
Fatigue	0 (0.0)	1 (2.9)	2 (5.9)
Headache	2 (5.9)	1 (2.9)	0 (0.0)
Nausea	2 (5.9)	0 (0.0)	0 (0.0)

The majority of adverse events reported during treatment were rated as mild or moderate in intensity. Five subjects, 3 in the darapladib 40-mg group and 2 in the darapladib 80-mg group, experienced a serious adverse event; however, none of these serious adverse events was considered to be related to study treatment.

## Discussion

This study demonstrated that short-term (14 days ±4 days) treatment with darapladib, a novel, selective Lp-PLA_2_ inhibitor, consistently produced a dose-dependent reduction in plasma Lp-PLA_2_ activity in patients with carotid artery disease. These data are consistent with several other published studies [15], [22]. However, this current study is the first to demonstrate that Lp-PLA_2_ activity within advanced human atherosclerotic lesions can be reduced (to a comparable extent as in plasma) following 14 days of treatment with darapladib. Darapladib was generally well tolerated in these studies, with no evidence of safety concerns.

Based on the demographic characteristics (mean age, body weight, medical history, and concomitant medication use) of the enrolled participants in this study, most patients had documented carotid disease. The degree of carotid artery stenosis (83.5%) in this carotid endarterectomy study suggests the presence of advanced atherosclerotic lesions. Nevertheless, these results indicate that darapladib distributed into lesions to produce its pharmacodynamic effect in a dose-dependent manner, with the 40-mg dose associated with a greater than 50% reduction in Lp-PLA_2_ activity and the 80-mg dose associated with an 80% reduction in activity compared with placebo. This demonstration of a pharmacodynamic effect of darapladib within atherosclerotic lesions is an important step in exploring whether Lp-PLA_2_ activity contributes directly to plaque destabilization and atherothrombosis and whether darapladib can modulate these processes to stabilize plaques and reduce CV risk. It is noteworthy that a similar level of plaque Lp-PLA_2_ activity inhibition was achieved with darapladib in a porcine model of coronary atherosclerosis, because in this model, Lp-PLA_2_ inhibition was accompanied by a significant reduction in complex plaque development [13]. Also noted in the porcine study with darapladib treatment was a 72% reduction in the coronary expression of MMP-9, which is in line with the dose-dependent decrease in MMP-9 mRNA observed in the current study and may suggest that plaque MMP-9 expression is downstream of Lp-PLA_2_ action. This is of potential significance because MMP-9 is greatly upregulated in culprit lesions and has been implicated in necrotic core expansion and plaque vulnerability [23]–[24].

Results from recent studies indicate that Lp-PLA_2_ staining/expression is most intense in lesions obtained from patients with advanced atherosclerosis (both coronary and carotid) [8]–[11]. In two independent studies, levels of lysoPC, a product of Lp-PLA_2_ activity, were found in significantly greater concentrations in symptomatic than in asymptomatic carotid plaques and were highly and significantly correlated with Lp-PLA_2_ levels [10]–[11]. Lp-PLA_2_ concentrations in symptomatic lesions were greatest within the necrotic core and in the shoulder regions, which were also regions known to have high concentrations of macrophages and oxidized low-density lipoprotein [8], [25].

In the current carotid endarterectomy study, no statistically significant differences were observed between treatment groups in plaque lysoPC levels (total or subfractions). Furthermore, no statistically significant differences were observed between treatment groups in serum biomarkers or in other plaque biomarkers, including Lp-PLA_2_ mRNA. However, a short treatment regimen (2 to 4 weeks) was utilized, which could account for these results. In a separate dose-ranging trial [21] involving a longer duration of treatment (12 weeks) and including a 160-mg dose of the enteric-coated formulation of darapladib that provides comparable exposure to the 80-mg dose of the non–enteric-coated formulation used in the present study, a significant reduction was observed in plasma IL-6 compared with placebo (P<0.028). No statistically significant changes were observed for other CV risk markers.

Although histopathologic assessment of carotid plaque composition was not performed on excised lesions, the excised plaques contained a surprisingly large amount of lysoPC (∼3 µmol/g of tissue). If lesion Lp-PLA_2_ was the primary source for the majority of this lysoPC, then the greatest benefit of darapladib would be dependent upon the clearance of this intra-lesional pool of proinflammatory lipid, which could conceivably take several weeks or even months of treatment to normalize. The reduction, however, in two markers of apoptosis, caspase-3 and caspase-8 activity, versus placebo and a correlation between Lp-PLA_2_ inhibition and caspase activity are intriguing findings. Apoptosis is increased in atherosclerotic lesions, with most cell death occurring within the macrophage-rich necrotic core of the plaque [26]. Although these measures of apoptosis were from a post-hoc analysis of stored tissue samples and would need to be reproduced in a prospective study, the reduction in these apoptosis markers would appear to be in agreement with results from two other studies involving the longer use of darapladib. In the Integrated Biomarker and Imaging Study (IBIS-2) [15], treatment with darapladib for 12 months halted necrotic core expansion as assessed by serial intravascular ultrasound examinations. These findings are consistent with observations from a second study involving a preclinical model of atherosclerosis (diabetic, hypercholesterolemic porcine model), in which treatment with darapladib inhibited the progression to complex coronary artery lesions [13]. Whether these changes will translate into differences in clinical outcomes awaits the results of the previously mentioned ongoing phase III trials, STABILITY and SOLID-TIMI 52.

### Limitations

This study does not address the potential clinical effects of Lp-PLA_2_ inhibition with respect to CV events. Given the relatively small sample sizes and the short duration of treatment (2 to 4 weeks), these results should be viewed as preliminary and hypothesis-generating. Nevertheless, demonstrating a pharmacodynamic effect within human atherosclerotic lesions provides a rationale, along with results from other trials, for studying the effects of treatment with darapladib in larger trials of longer duration.

## Conclusions

Although it is widely accepted that intensifying treatment for primary and secondary prevention of CV disease improves outcomes, CV event rates for high-risk individuals remain unacceptably high. Treatments that modulate processes involved in plaque destabilization and atherothrombosis may offer additional benefit for reducing CV events. The findings from this study suggest that darapladib does indeed distribute into advanced human atherosclerotic lesions to produce a dose-dependent reduction in Lp-PLA_2_ activity that is comparable in magnitude to what has been observed in plasma. Results from ongoing event-driven trials are now needed to explore the effects of Lp-PLA_2_ inhibition on CV outcomes.

## Supporting Information

Appendix S1 **Study Investigators.**
(DOCX)

Checklist S1 **CONSORT Checklist.**
(PDF)

Protocol S1 **Study Identifier: SB-480848/010.** A multi-centre, randomised, double-blind, placebo-controlled, parallel-group study to investigate the effect of the Lp-PLA2 inhibitor SB-480848 (40, 80 mg od) on carotid plaque composition in patients with carotid artery disease and planned carotid endarterectomy, stratified for statin use and gender, after 14±4 days of treatment.
(PDF)
