# Supplementary material for: Effect of Darapladib Treatment on Endarterectomy Carotid Plaque Lipoprotein-Associated Phospholipase A2 Activity: A Randomized, Controlled Trial
Source: PLoS One. 2014 Feb 20;9(2):e89034. doi: 10.1371/journal.pone.0089034 (PMC3930668; doi:10.1371/journal.pone.0089034)
Supplement: Appendix S1 — Study Investigators. (DOCX) [file pone.0089034.s001.docx]

**STUDY INVESTIGATORS**

The following investigators, listed by country and the number of enrolled subjects included in the analyses, participated in Study 480848/010:

**Finland:** Markku Kaste, MD, PhD (5); Juhani Sivenius, MD, PhD (5); Matti Hillbom, MD, PhD (1)

**France:** Patrick Feugier, MD (3)

**Germany:** Michael Görtler, MD (9); Jörg Glahn, MD (3); Hans-Jörg Schütz (3); Andreas Hartmann, MD (2)

**The Netherlands:** Frank L. J. Visseren, MD, PhD (9); Frans L. Moll, MD, PhD (1)

**Poland:** Walerian Staszkiewicz, MD (22)

**Spain:** José Álvarez Sabín, MD, PhD (11); Angel Chamorro, MD (7); Francisco Rubio, MD (5); Ricardo Navarro, MD (4); José Castillo, MD (3); José-Luis Martí-Vilalta, MD (1)

**United Kingdom:** Lasantha Dinesh Wijesinghe, MB BChir, FRCS (6); Roger Baird, ChM (2)
