# Supplementary material for: Effect of Darapladib Treatment on Endarterectomy Carotid Plaque Lipoprotein-Associated Phospholipase A2 Activity: A Randomized, Controlled Trial
Source: PLoS One. 2014 Feb 20;9(2):e89034. doi: 10.1371/journal.pone.0089034 (PMC3930668; doi:10.1371/journal.pone.0089034)
Supplement: Protocol S1 — Study Identifier: SB-480848/010. A multi-centre, randomised, double-blind, placebo-controlled, parallel-group study to investigate the effect of the Lp-PLA2 inhibitor SB-480848 (40, 80 mg od) on carotid plaque composition in patients with carotid artery disease and planned carotid endarterectomy, stratified for statin use and gender, after 14±4 days of treatment. (PDF) [file pone.0089034.s003.pdf]

**Division:**	New Product Development	**Document Number:**	HM2003/00077/04
**Document Type:**	Protocol	**Study Identifier:**	SB480848/010
**Site of Issue:**	Harlow	**Protocol Amendment No.:**	Protocol Amendment No. 4
**Classification:**	Level 2	**Document Date:**	10 March 2003

Title:

A Multi-centre, randomised, double-blind, placebo-controlled, parallel-group study to investigate the effect of the Lp-PLA2 inhibitor SB-480848 (40,80mg od) on carotid plaque composition in patients with carotid artery disease and planned carotid endarterectomy, stratified for statin use and gender, after 14+/-4 days treatment.

Abstract: (For Internal Use Only)

Authors: Anne Muir

Compound Numbers/Keywords (if applicable):

SB480848

Distribution: *denotes summary only, **denotes partial copy only

Confidential

SB-480848

A multi-centre, randomised, double-blind, placebo-controlled, parallel-group study to investigate the effect of the Lp-PLA2 inhibitor SB-480848 (40, 80mg od) on carotid plaque composition in patients with carotid artery disease and planned carotid endarterectomy, stratified for statin use and gender, after 14+/-4 days treatment

010

Anne Muir BSc (Hons)*

Clinical Development and Medical Affairs, (Europe)

Dr Ameet Nathwani, MRCP; **Vice President, Clinical Development and Medical Affairs CV and Metabolic, Europe;** Dr Tony G Johnson, MD **Vice President, Discovery Medicine**

GSK Document Number: HM2003/00077/04

Date signed of 10th March 03

Incorporating Protocol amendment 1, Protocol amendment 2, Protocol amendment 3 and Protocol amendment 4.

SPONSOR INFORMATION PAGE

Title: A Multi-centre, randomised, double-blind, placebo-controlled, parallel-group study to investigate the effect of the Lp-PLA2 inhibitor SB-480848 (40,80mg od) on carotid plaque composition in patients with carotid artery disease and planned carotid endarterectomy, stratified for statin use and gender, after 14+/-4 days treatment.

Study Identifier: SB-480848/010

Sponsor Contact Information:

GlaxoSmithKline

New Frontiers Science Park

Third Avenue

Harlow, Essex CM19 5AW, UK

Telephone: +44 (0) 1279 622 100

INVESTIGATOR PROTOCOL AGREEMENT PAGE

I agree:

- To assume responsibility for the proper conduct of the study at this site.
- To conduct the study in compliance with this protocol, any future amendments, and with any other study conduct procedures provided by GlaxoSmithKline (GSK).
- Not to implement any changes to the protocol without agreement from the sponsor and prior review and written approval from the Institutional Review Board (IRB) or Independent Ethics Committee (IEC), except where necessary to eliminate an immediate hazard to the subjects, or for administrative aspects of the study (where permitted by all applicable regulatory requirements).
- That I am thoroughly familiar with the appropriate use of the investigational product(s), as described in this protocol, and any other information provided by the sponsor including, but not limited to, the following: the current Clinical Investigator's Brochure / Investigator's Brochure (CIB/IB) or equivalent document, CIB/IB supplement (if applicable), and approved product label (if the product is marketed in this country and the label is not already provided as an equivalent to a CIB/IB).
- That I am aware of, and will comply with, "good clinical practices" (GCP) and all applicable regulatory requirements.
- To ensure that all persons assisting me with the study are adequately informed about the GSK investigational product(s) and of their study-related duties and functions as described in the protocol.
- That I have been informed that certain regulatory authorities require the Sponsor to obtain and supply, as necessary, details about the investigator's ownership interest in the Sponsor or the investigational product, and more generally about his/her financial ties with the Sponsor. GSK will use and disclose the information solely for the purpose of complying with regulatory requirements.

Hence I:

- Agree to supply GSK with any necessary information regarding ownership interest and financial ties (including those of my spouse and dependent children);
- Agree to promptly update this information if any relevant changes occur during the course of the study and for 1 year following completion of the study; and
- Agree that GSK may disclose any information it has about such ownership interests and financial ties to regulatory authorities.

Investigator Name:

Investigator Signature ______________________________ Date ______________

TABLE OF CONTENTS

PAGE

ABBREVIATIONS....9

PROTOCOL SUMMARY 11

1. INTRODUCTION 13
   1.1. Background....13
   1.2. Rationale for Study....17
   1.3. Rationale for Dose 17

2. OBJECTIVE(S)....18
   2.1. Primary 18
   2.2. Secondary....18

3. ENDPOINT(S) 19
   3.1. Primary 19
   3.2. Secondary....19

4. STUDY DESIGN....19

5. STUDY POPULATION 20
   5.1. Number of Patients 20
   5.2. Eligibility Criteria 20
      5.2.1. Inclusion Criteria 20
      5.2.2. Exclusion Criteria 21
      5.2.3. Other Eligibility Criteria Considerations 21
      5.2.4. Pharmacogenetics 22

6. STUDY ASSESSMENTS AND PROCEDURES 22
   6.1. Demographic and Baseline Assessments 22
      6.1.1. Demography 22
      6.1.2. Medical history/medication 22
      6.1.3. Physical examination 23
   6.2. Safety....23
      6.2.1. Vital signs 23
      6.2.2. Electrocardiogram (ECG) 23
      6.2.3. Chest X-ray....23
      6.2.4. Clinical measurements....24
         6.2.4.1. Haemoglobin A1c, Insulin 24
         6.2.4.2. Peripheral Lymphocytes 24
         6.2.4.3. Haematology and Clinical Chemistry 24
      6.2.5. Pregnancy....24
         6.2.5.1. Pregnancy Testing....25
         6.2.5.2. Time Period for Collecting Pregnancy Information....25
         6.2.5.3. Action to be Taken if Pregnancy Occurs 25
      6.2.6. Pharmacogenetic sampling 25

6.3. Efficacy 26
6.3.1. Primary Efficacy 26
6.3.1.1. Plaque Lp-PLA2 Activity 26
6.3.2. Secondary....................26
6.3.2.1. Plasma Lp-PLA2 activity 26
6.3.2.2. Plasma and plaque Lp-PLA2 mass 27
6.4. Biomarker(s) 27
6.5. Pharmacokinetics and Pharmcodynamics 27
6.5.1. PK/PD Sampling Schedule....................27

7. INVESTIGATIONAL PRODUCT(S) 29
7.1. Description of Investigational Product 29
7.2. Dosage and Administration 29
7.3. Dose Rationale 29
7.3.1. Pharmacogenetics 30
7.4. Blinding 30
7.5. Treatment Assignment 30
7.6. Packaging and Labeling....................31
7.7. Preparation 32
7.8. Handling and Storage 32
7.9. Product Accountability....................32
7.10. Assessment of Compliance....................32
7.11. Treatment of Investigational Product Overdose....................33
7.12. Occupational Safety 33

8. CONCOMITANT MEDICATIONS AND NON-DRUG THERAPIES....................33
8.1. Permitted Medications....................33
8.2. Prohibited Medications....................33

9. PATIENT COMPLETION AND WITHDRAWAL 34
9.1. Patient Completion....................34
9.2. Patient Withdrawal 34
9.2.1. Patient Withdrawal from Study 34
9.2.2. Patient Withdrawal from Investigational Product 34
9.3. Screen and Baseline Failures 35

10. ADVERSE EVENTS (AE) AND SERIOUS ADVERSE EVENTS (SAE) 35
10.1. Definition of an AE 35
10.2. Definition of a SAE 36
10.2.1. Surgery-Related Events or Outcomes Not Qualifying as SAEs....................37
10.3. Lack of Efficacy....................37
10.4. Clinical Laboratory Abnormalities and Other Abnormal Assessments as AEs and SAEs....................37
10.5. Time Period, Frequency, and Method of Detecting AEs and SAEs 38
10.6. Recording of AEs and SAEs 38
10.7. Evaluating AEs and SAEs....................39
10.7.1. Assessment of Intensity 39
10.7.2. Assessment of Causality....................39
10.8. Follow-up of AEs and SAEs 40
10.9. Prompt Reporting of SAEs to GSK....................40
10.9.1. Timeframes for Submitting SAE Reports to GSK 40

10.9.2. Completion and Transmission of the SAE Reports......41
10.10. Regulatory Reporting Requirements for SAEs......41
10.11. Post-Study AEs and SAEs......42
10.12. SAEs Related to Study Participation......42

11. DATA ANALYSIS AND STATISTICAL CONSIDERATIONS......42
11.1. Hypotheses......42
11.2. Treatment Comparisons of Interest......42
11.2.1. Primary Comparisons of Interest......42
11.2.2. Other Comparisons of Interest......43
11.3. Interim Analysis......43
11.4. Sample Size Considerations......43
11.4.1. Sample Size Assumptions......43
11.4.2. Sample Size Sensitivity......43
11.4.3. Sample Size Re-Estimation......44
11.5. Analysis Populations......44
11.5.1. Data Sets......45
11.6. General Considerations for Data Analysis......45
11.6.1. Withdrawal......45
11.6.2. Missing Data......45
11.6.3. Derived and Transformed Data......45
11.6.4. Assessment Windows......45
11.6.5. Other Issues......46
11.7. Efficacy Analysis......46
11.7.1. Primary Analysis......46
11.7.2. Secondary Analysis......46
11.7.3. Other Efficacy Analysis......47
11.8. Safety Analysis......47
11.8.1. Extent of Exposure......47
11.8.2. Adverse Events......48
11.8.3. Clinical Laboratory Evaluations......48
11.8.4. Other Safety Measures......48
11.8.4.1. Vital Signs......48
11.8.4.2. ECGs......49
11.9. Biomarker(s) Analyses......49
11.10. Clinical Pharmacology Data Analyses......49
11.10.1. Pharmacokinetic Analyses......49
11.10.2. Pharmacodynamic Analyses......49
11.10.3. Pharmacokinetics/Pharmacodynamics Analyses......49
11.11. Pharmacogenetic analysis......50
11.11.1. Scope of Phamacogenetic Analysis......50
11.11.2. Coded sample......50

12. STUDY ADMINISTRATION......51
12.1. Regulatory and Ethical Considerations......51
12.1.1. Regulatory Authority Approval......51
12.1.2. Ethical Conduct of the Study and Ethics Approval......51
12.1.3. Informed consent......51
12.1.4. Investigator Reporting Requirements......52
12.2. Study Monitoring......52
12.3. Quality Assurance......52
12.4. Study and Site Closure......53

12.5. Records Retention 53
12.6. Provision of Study Results and Information to Investigators 54
12.7. Information Disclosure and Inventions....54
12.8. Data Management 56
12.8.1. Pharmacogenetics 56

13. REFERENCES....57

14. APPENDICES 59
14.1. Appendix 1 Time and Events Table....59
14.2. Appendix 2 Declaration of Helsinki....61
14.3. Appendix 3 Classification of Unstable Angina 66
14.4. Appendix 4 Country Specific Requirements 67

Abbreviations

AE	Adverse Experience/Event
AUC	Area Under Curve
BMI	Body Mass Index (weight (kg)/square of height (m^2))
BPM	Beats per Minute
CD40L	CD40 ligand
CEA	Carotid Endarterectomy
CHD	Coronary Heart Disease
CRF	Case Report Form
hsCRP	High Sensitivity C Reactive Protein
CV	Cardiovascular
CVD	Cardiovascular Disease
dL	Decilitre
ECG	Electrocardiogram
GCP	Good Clinical Practice
GSK	GlaxoSmithKline
HbA1c	Glycated Haemoglobin
HDL	High Density Lipoprotein
HDLc	High Density Lipoprotein Cholesterol
ICH	International Conference on Harmonisation
IEC	Independent Ethics Committee
IL-6	Interleukin-6
IRB	Institutional Review Board
IV	Intravenous
L	Litre
LDL	Low Density Lipoprotein
LDLc	Low Density Lipoprotein Cholesterol
Lp-PLA_2	Lipoprotein-activated Phospholipase A2
mg	Milligrams
min	Minute
mL	Millilitre
mmol/L or mM	Millimolar
MMP-9	Matrix Metalloproteinase-9
MRI	Magnetic Resonance Imaging
Msec	Millisecond
PAF	Platelet Activating Factor
PAF-AH	Platelet Activating Factor-Acetyl Hydrolase
PAI-1	Plasminogen Activator Inhibitor Type 1
PD	Pharmacodynamic
PK	Pharmacokinetic
PoC	Proof of Concept
RAP	Reporting and Analysis Plan
SAE	Serious Adverse Experience/Event
sICAM	soluble Intercellular Adhesion Molecule

TC	Total Cholesterol
TIA	Transient Ischaemic Attack
μ	Micro
UK	United Kingdom
μL	Microlitre
μmol	Micromole
WBC	White Blood Cell
WHO	World Health Organisation
WRC-GCP	Worldwide Regulatory Compliance – Good Clinical Practice

Protocol Summary

Rationale

SB-480848, an active inhibitor of lipoprotein phospholipase A2 (Lp-PLA_2), may prevent the accumulation of lyso-PC and other pro-inflammatory lipids generated during the oxidation of LDL. Lyso-PC has several pro-atherogenic activities ascribed to it, including monocyte chemotaxis and induction of endothelial dysfunction, both of which facilitate monocyte-derived macrophage accumulation within the artery wall. As the approach specifically targets the underlying chronic inflammation, an inhibitor of Lp-PLA_2 is predicted to stall intimal macrophage accumulation and therefore retard and stabilise the developing atherosclerotic plaque.

To date, it has been demonstrated that single and repeat oral doses of SB-480848 significantly inhibit plasma Lp-PLA_2 activity in human volunteers. In addition, SB-480848 has shown *in vivo* inhibition of Lp-PLA_2 in atherosclerotic plaque of WHHL rabbit aortae.

The next step in establishing proof-of-mechanism, is to demonstrate that SB-480848 inhibits Lp-PLA_2 activity in human carotid plaques *in vivo*. In this study, patients with planned carotid endarterectomy for the treatment of carotid artery disease will take SB-480848 (40mg or 80mg), or matching placebo, for 14+/-4 days prior to surgery. The excised plaques will be analysed for Lp-PLA_2 and relevant cardiovascular biomarkers, and compared to plasma levels.

Objective(s)

The primary objective is to determine Lp-PLA_2 activity in atherosclerotic carotid plaques after 14(+/-4) days treatment with SB-480848, compared to placebo. Secondary objectives include determination of the change in Lp-PLA_2 activity in blood, Lp-PLA_2 mass in blood and plaque, specified biomarkers in blood and plaque and their respective correlation's with Lp-PLA_2. In addition, the study aims to characterise the PK/PD of repeat oral doses of SB-480848, and safety and tolerability in this population.

Endpoint(s)

The primary endpoint is Lp-PLA_2 activity in atherosclerotic plaque removed during carotid endarterectomy. Secondary endpoints include Lp-PLA_2 activity in blood, Lp-PLA_2 mass in blood and plaque, and specified biomarkers in blood and plaque.

Study Design

A multi-centre, randomised, double-blind, placebo controlled, parallel group study in patients with documented carotid artery disease requiring surgery. Eligible patients will take study medication (40mg SB-480848, 80mg SB-480848, or matching placebo) for 14+/- 4 days prior to planned surgery.

Study Population

Male or female patients (post-menopausal or using adequate contraception), 35 years of age or above, with planned carotid endarterectomy will be eligible for the study. Ninety-nine (99) patients will be randomised to study medication.

Study Assessments and Procedures

At the screening visit, informed consent and eligibility assessments (including vital signs, clinical chemistry, haematology, chest X-ray if applicable) will be performed. Up to a maximum of 4 weeks later, an eligible patient will be randomised to one of 2 treatment groups (40mg, 80mg) or matching placebo. An ECG will be performed at Visits 2 and 5. Study medication will be taken once daily for 14 +/- 4 days. Patients will attend for one out-patient visit during their study medication treatment period. The end of treatment will coincide with planned carotid endarterectomy and patients will take their last dose of study medication on the day prior to surgery. Patients will attend for a follow-up visit 2 weeks after the last dose of study medication.

Fasting blood samples will be taken at 4 timepoints during the study for the analysis of specified biomarkers. Safety bloods (clinical chemistry, haematology) will be performed at 5 visits.

Investigational Product(s)

At Visit 2 (baseline) patients will be randomised on a 1:1:1 basis to receive SB-480848 40mg, 80mg or matching placebo. Patients will be given 2 bottles of study medication and will be instructed to take 2 tablets from each bottle once daily. Study medication will be taken by patients for 14+/-4 days prior to planned carotid endarterectomy surgery.

1. INTRODUCTION

SB-480848 is a novel, selective and orally active inhibitor of lipoprotein-associated phospholipase A2 (Lp-PLA_2) and has potential for the treatment of atherosclerosis.

Lp-PLA_2 is often referred to in the literature as platelet-activating factor acetyl hydrolase (PAF-AH) since it was first characterised as a plasma enzyme activity that hydrolysed exogenously added PAF. Lp-PLA_2 and PAF-AH are different names for the same enzyme but Lp-PLA_2 is the more general term because this enzyme can hydrolyse a broad range of substrates in addition to PAF.

1.1. Background

Cardiovascular (CV) disease accounts for approximately 50% of all deaths (from myocardial infarction and stroke) in both developed and developing countries. [ROSS, 1993] Atherosclerosis is the fundamental pathology behind most CV deaths and is now universally acknowledged as an inflammatory disease and not one that simply results from the arterial accumulation of lipids. This specialised inflammatory disease of the intima is characterised by leukocyte (monocytes and T-lymphocytes) recruitment and accumulation having a major role in both early and continuing aspects of its pathogenesis. Indeed, the histopathological examination of plaques that have been identified as culprit lesions causing a myocardial infarction, have a soft lipid core and a thin friable cap with a high concentration of macrophages in the so called "shoulder region." Thus, macrophage driven chronic inflammation appears to represent an important destabilising process in the arterial lesion [ROSS, 1993].

The sub-endothelial oxidation of low density lipoprotein (LDL) is viewed as a highly significant biological process that both initiates and accelerates arterial lesion development. [ROSS, 1993] One of the earliest events in LDL oxidation is the hydrolysis of oxidatively modified phosphatidylcholine, generating substantial quantities of lysophosphatidylcholine (lyso-PC) and oxidised fatty acids. This hydrolysis is mediated solely by lipoprotein-associated phospholipase A_2 (Lp-PLA_2, also known as PAF-AH), an enzyme that is found associated predominantly with LDL in human plasma [TEW DG, 1996]. Lp-PLA_2 is a subtype of the growing phospholipase A_2 superfamily with a broad substrate preference because it can hydrolyse PAF as well as more complex polar phosphatidylcholines. A significant volume of evidence has accumulated in favour of both lyso-PC and oxidised fatty acids, being proinflammatory and proatherogenic mediators [MACPHEE, 1999]. Thus, preventing the generation of these mediators through inhibition of Lp-PLA_2 should retard atherosclerosis by interfering with inflammatory cell localisation, activation, pro-inflammatory function and death.

At present, very little is known about the basic epidemiology of Lp-PLA_2. In the largest study to date (by WOSCOPS researchers [PARKARD 2000], levels of Lp-PLA_2 had a strong, positive association with risk that was not confounded by other CVD risk factors. It was associated with almost a doubling of the risk of coronary events (non-fatal myocardial infarction, death from CHD or a revascularisation procedure) in the highest

quintile as compared with the lowest quintile. However, the generalizability of these data is limited since they are based on a single, homogeneous European population sample. Thus, at this time, there is a need to broaden the epidemiological studies of Lp-PLA_2 to include larger, more diverse populations to test if the association of Lp-PLA_2 with risk factors and cardiovascular risk continues to hold. GSK has therefore developed an ongoing epidemiology programme which aims to clearly depict the relationship between Lp-PLA_2 and cardiovascular disease outcomes in different populations.

SB-480848, an active inhibitor of lipoprotein phospholipase A2 (Lp-PLA_2), may prevent the accumulation of lyso-PC and other pro-inflammatory lipids generated during the oxidation of LDL. Lyso-PC has several pro-atherogenic activities ascribed to it, including monocyte chemotaxis and induction of endothelial dysfunction, both of which facilitate monocyte-derived macrophage accumulation within the artery wall. As the approach specifically targets the underlying chronic inflammation, an inhibitor of Lp-PLA_2 is predicted to stall intimal macrophage accumulation and therefore retard and stabilise the developing atherosclerotic plaque.

Nonclinical studies carried out to support the clinical protocol include primary pharmacology studies demonstrating inhibition of Lp-PLA_2. The pharmacokinetics of SB-480848 have been investigated in relevant nonclinical species. The toxicity of SB-480848 has been studied following repeat oral administration for up to 4 weeks in rats and dogs, and reproductive toxicity studies and *in vitro* and *in vivo* genotoxicity tests have also been completed.

SB-480848 has shown *in vitro* inhibition of Lp-PLA_2 in plasma (WHHL rabbit and human) and atherosclerotic plaque (WHHL rabbit aorta). Other *in vitro* assays have demonstrated that SB-480848 inhibits lyso-PC generation during the oxidation of human LDL and prevents the generation of monocyte chemoattractants during LDL oxidation. SB-480848 was orally active in the WHHL rabbit as evidenced by *in vivo* inhibition of Lp-PLA_2 in plasma and atherosclerotic plaque.

Two studies were conducted to investigate the effect of SB-480848 on plaque vs plasma Lp-PLA_2 inhibition and on plaque size in old WHHL rabbits with established plaque or on plaque development in young WHHL rabbits in which lesions were growing rapidly. Reporting of these studies is ongoing. Old male WHHL rabbits (12-14 months old) were dosed by gavage for 28 days with either 0.5, 2.0 or 8.0 mg/kg/day SB-480848 or vehicle alone. There were no significant differences in lesion volume or cross-sectional area.

In a second, longer-term study young male WHHL rabbits (12 weeks old) were treated with either SB-480848 (30 mg/kg/day) in the diet or given diet alone for 16 weeks. Blood was taken throughout the study, the rabbits killed, aortae removed and atherosclerosis measured by *ex-vivo* MRI, en face, and by histology. Histological sections were stained with haematoxylin and eosin for detailed microscopic examination. The rate of decline in plasma cholesterol was reduced in rabbits treated with SB-480848 so that from Week 8 onwards it was significantly higher (15%-20%) in treated versus control rabbits.

Lesion volume measured *ex-vivo* by MRI and lesion area en face were both unaffected by treatment. However, the lesion cross-sectional area in one segment was significantly larger in SB-480848-treated rabbits than in controls. Macrophage area within the lesions

was also increased by treatment in 2 segments. However, as the increase was in proportion to the size of the lesions, there was no effect of SB-480848 on the percentage macrophage area.

The increase in lesion areas may have been caused by the increased exposure to plasma cholesterol that resulted from the decrease in the rate of decline in plasma cholesterol seen in SB-480848-treated rabbits. When terminal cholesterol was used as a covariate in the statistical analysis of lesion areas, the differences in lesion (or macrophage) area with treatment was not statistically significant. The effect of SB-480848 on plasma cholesterol was unexpected. No effect of treatment was seen in old WHHL rabbits treated for 28 days with SB-480848 as shown above or in dogs or rats given 100 mg/kg/day by oral gavage for 28 days in the repeat-dose toxicity studies [BUSS N, 2001; FREEMAN L, 2001]. One important difference between the young and older WHHL rabbits is that, as in humans, plasma cholesterol is normally stable with time in old WHHL rabbits but declines rapidly in young rabbits. It is probable that the effect of SB-480848 on the rate of decline of plasma cholesterol after 8 weeks of treatment and, possibly, through this on atherosclerosis in young WHHL rabbits is due to subtle changes in lipoprotein metabolism in this severely hyperlipidaemic model of familial hypercholesterolaemia. When the data from the young WHHL rabbits is put into context with the results from the dog, rat and older WHHL rabbit studies, there is little evidence to suggest that SB-480848 will have significant effects on plasma cholesterol in humans during a 28-day treatment period.

In single-dose pharmacology studies in conscious male rats, SB-480848 was associated with small, transient increases in body temperature (10, 30 and 100mg/kg) and minor, transient decreases in respiratory rate (≥10mg/kg) accompanied at 100mg/kg by small, transient increases in mean arterial pressure and a mild to moderate, reversible decrease in pulmonary ventilation. Doses ≥30mg/kg induced a transient decrease in urine osmolality associated with a dose-dependent increase in water intake, and at 100mg/kg, there was a slight increase in the rate of urea excretion and a slight decrease in urinary pH.

Pharmacokinetic, absorption, distribution, metabolism and elimination studies have been performed in rats and dogs, the species selected for the toxicological evaluation of SB-480848. In addition, the pharmacokinetics of SB-480848 have been investigated in the rabbit and monkey, and the *in vitro* metabolism of SB-480848 has been investigated in hepatocytes from various preclinical species and humans to validate the use of these species in assessing the safety of SB-480848 for administration to humans via the oral route.

The data obtained on the disposition of SB-480848 to date support the use of rats and dogs as relevant species for the evaluation of the safety of SB-480848 for clinical use. Systemic exposures in these species following repeat oral administration of SB-480848 were generally substantially greater than those predicted to occur in humans at the maximum proposed dose (80mg; equivalent to 1.6 mg/kg based on a 50kg person). The available metabolism data indicate that all metabolic pathways apparent in human hepatocytes were also seen in rats and dogs. Therefore, there are no findings that would

preclude the administration of SB-480848 via the oral route to patients in accordance with the proposed clinical protocol.

Following repeat oral administration of SB-480848 for up to 28 days, the principal findings in rats of intestinal irritancy at ≥100 mg/kg/day and phospholipid accumulation at ≥30 mg/kg/day were reversible within 1 month after cessation of dosing. In dogs, foamy macrophage accumulation in the mesenteric lymph nodes was evident at ≥30 mg/kg/day but was not characteristic of phospholipid accumulation. There were no toxicologically important findings in rats and dogs given 10 mg/kg/day for 1 month.

SB-480848 had no effect on mating, fertility or gonadal function at any dose in male or female rats and no effect on embryofoetal development in the rabbit. In the embryofoetal development study in rats, a low incidence of rudimentary ribs was observed at 100 mg/kg/day (at ratios of 13.4- and 31.0-fold higher than the respective AUC and C_{max} exposures in humans at the proposed upper clinical dose). Since women of childbearing potential may be included in the proposed study, appropriate contraceptive measures should be followed.

SB-480848 showed no evidence of genotoxic activity in a range of *in vitro* and *in vivo* assays.

Systemic exposures (AUC and C_{max}) achieved during the repeat-dose toxicity studies in rats and dogs were generally higher than exposure in humans at the upper dose proposed for use in the clinical study, although only equivalent or modestly higher at the no effect level in animals (1.0-to 2.4-fold for AUC and 3.9- to 7.8-fold for C_{max}). However, given the nature of the findings and the fact that the mild phospholipid accumulation in rats was reversible, it is considered that there are no toxicological implications that would preclude the oral administration of SB-480848 to humans under the conditions of the proposed protocol for the clinical study. As a precaution in this study, blood samples collected at Visits 2 and 5 will be examined for peripheral lymphocytes by electron microscopy, to monitor phospholipid accumulation.

By the end of July 2002, a total of 156 healthy subjects received at least one dose of SB-480848 (dose range 0.5 to 100 mg). Single oral doses of SB-480848 were received by 114 subjects. Of these, 23 subjects also received repeat oral doses of SB-480848 in the 002 study. [FINNERTY] Repeat oral doses of SB-480848 alone and with atorvastatin have been administered to 42 male subjects in study 007. [FINNERTY] SB-480848 is readily absorbed from the gastrointestinal tract. The half life of the compound is approximately 25-45 hours and steady state is achieved in approximately 10 days. SB-480848 is N-deethylated to SB-553253 that is quantifiable in human plasma. SB-480848 has been generally safe and well tolerated. There have not been any serious adverse events (SAEs) reported to date. The most common treatment emergent adverse event (AE) across studies was headache.

Atorvastatin is a lipid-lowering agent metabolized by cytochrome P450 enzyme 3A4 and is a potential concomitant medication in the target population of SB-480848. The pharmacokinetics of SB-480848 and atorvastatin when administered alone and in combination for 10 days were investigated in a parallel group study in 34 healthy adult male subjects (SB-480848/RSD-101PS9/3).

AUC (0-24) and C_{max} of SB-480848 were decreased when given with atorvastatin compared to given alone; however, the decreases were on average less than 20%. With co-administration of atorvastatin, there were also decreases (approximately 35%) in the C_{max} and AUC of SB-553253 (the N-desethylated metabolite of SB480848). Since this metabolite is not believed to contribute to the activity of SB-480848 due to its low exposure and potency relative to SB-480848, the decreased exposure is not expected to have any clinical relevance.

Co-administration of SB-480848 had no effect on AUC(0-24) of atorvastatin with only small decrease (on average 16%) in C_{max}. The minor differences observed in the pharmacokinetics of SB-480848 and atorvastatin are not considered to be clinically relevant and therefore, based on the results of this study, SB-480848 may be co-administered with atorvastatin.

Ongoing enzymology studies aim to provide clarification that SB-480848 does not induce CYP 3A4. Until results are available, it is unclear whether SB-480848 may have an effect on oestrogen. Therefore, as a pre-cautionary measure, female patients of childbearing potential should use an alternative or additional contraceptive method to the oral contraceptive pill.

Please refer to the Investigator's Brochure for SB-480848 for full details of pre-clinical and clinical data.

1.2. Rationale for Study

To date, it has been demonstrated that single and oral doses of SB-480848 significantly inhibit plasma Lp-PLA_2 activity in human volunteers. In addition, SB-480848 has shown *in vivo* inhibition of Lp-PLA_2 activity in atherosclerotic plaque of WHHL rabbit aortae.

The next step in establishing proof-of-mechanism, is to demonstrate that SB-480848 inhibits Lp-PLA_2 activity in human carotid plaques *in vivo*. In this study, patients with planned carotid endarterectomy for the treatment of carotid artery disease will take SB-480848 (40mg or 80mg), or matching placebo, for 14+/-4 days prior to surgery. The excised plaques will be analysed for Lp-PLA_2 and relevant cardiovascular biomarkers, and compared to plasma levels.

1.3. Rationale for Dose

Patients will be randomised in a 1:1:1 ratio to receive either 40mg SB-480848 once daily or 80mg SB-480848 once daily or matching placebo once daily. Single oral doses of up to 100mg have been safe and well tolerated in the 001 study [SHADDINGER]. Repeat doses of up to 80mg have been safe and well tolerated in the 002 study [FINNERTY].

PK/PD modelling has been undertaken to assess the dose/drug concentration/plasma Lp-PLA_2 enzyme activity inhibition relationship for SB-480848. Modelling and simulation suggest that peak and trough inhibition of plasma Lp-PLA_2 enzyme activity appear to begin to plateau at doses around 80 mg. It is anticipated that once daily doses of 80 and 40 mg SB-480848 are likely to result in approximately 85-90% and 60-65% trough (24-

hour post-dose) plasma Lp-PLA_2 enzyme inhibition at steady state, respectively. The level of correlation between plaque and plasma enzyme inhibition is not known, however, this will be investigated in the proposed study.

Modelled data also suggests that steady-state of drug concentration and plasma Lp-PLA_2 enzyme inhibition is achieved following 10 days once daily dosing of SB-480848 (half life in man is 25-45 hours). Preclinical data showed significant plaque enzyme inhibition two hours post-dose in Watanabe heritable hyperlipidaemic (WHHL) rabbits following SB-480848 dosing via gavage. This preclinical observation suggests that distribution of SB-480848 into plaque is not a rate-limiting step in the rabbit model. A treatment duration of 14± 4 days is therefore selected in this study.

2. OBJECTIVE(S)

2.1. Primary

- Lp-PLA2 activity in atherosclerotic carotid plaques after 14(+/-4) days treatment with SB-480848, compared to placebo

2.2. Secondary

- To determine the change in Lp-PLA2 activity in blood after treatment with SB-480848
- To determine the change in Lp-PLA2 mass in blood after treatment with SB-480848
- Lp-PLA2 mass in plaque after treatment with SB-480848, compared with placebo
- To evaluate the dose response effects of SB-480848 on plasma and plaque Lp-PLA2 levels
- Levels of defined biomarkers in plaque after treatment with SB-480848, compared with placebo
- To determine the change in levels of defined biomarkers in blood after treatment with SB-480848
- To investigate the correlation between biomarkers and Lp-PLA2 in blood and plaque after treatment with SB-480848
- To characterise the pharmacokinetics (PK) of repeat oral doses of SB-480848 and its metabolite (SB-553253) in this patient population
- To investigate the pharmacodynamics (PD) of SB-480848 following repeat oral administration to this population
- To further investigate the safety and tolerability of SB-480848 through observations of adverse experiences, vital signs, laboratory tests, and effects on 12-lead ECG.
- To determine a change in the oxidised lipids and their metabolites including lyso-PC levels in plasma and in carotid plaques after 14(+/-) days treatment with SB-480848 compared to placebo.

Pharmacogenetic

- To determine the variants of candidate genes associated with clinical response to SB-480848
- To determine the variants of candidate genes associated with the absorption, metabolism, distribution and excretion of SB-480848
- To determine the variants of candidate genes associated with tolerability or adverse events experienced during administration of SB-480848

3. ENDPOINT(S)

3.1. Primary

- Activity of Lp-PLA2 in atherosclerotic plaque removed during carotid endarterectomy

3.2. Secondary

- Mass of Lp-PLA2 in plaque removed during carotid endarterectomy
- Activity and mass of Lp-PLA2 in blood
- Concentrations of the following markers in the plasma/serum:
  Total cholesterol; high density lipoprotein (HDL); low density lipoprotein (LDL); triglycerides; high sensitivity C-reactive protein (hsCRP); CD40 ligand (CD40L); soluble intracellular adhesion molecules (sICAM); E-selectin; plasminogen activating inhibitor type 1 (PAI-1) activity and antigen; matrix metalloproteinase-9 (MMP-9), oxidised lipids and their metabolites including lyso-PC.
- Expression and/or concentration of the following markers in excised plaques:
  CD68 (Macrophages); CD3 (T cells); CD20 or 22 (B cells); α-actin (smooth muscle cells); CD40L; MMP -2 and -9; PAI –1; ICAM; IL-6; Lp-PLA2 (mRNA expression, oxidised lipids and their metabolites including lyso-PC.

4. STUDY DESIGN

A multi-centre, randomised, double-blind, placebo controlled, parallel group study in patients with documented carotid artery disease requiring surgery.

Male or female patients (post-menopausal or using adequate contraception), 35 years of age or above, with planned carotid endarterectomy will be eligible for the study.

Patients will be required to attend the hospital as an out-patient approximately 4 times for study-related procedures. Additionally, study procedures will be conducted on 3 occasions during a patient's planned hospital stay for carotid endarterectomy.

The study consists of 3 parts:

1. **Screen** - at Visit 1, written, informed consent and eligibility assessments will be performed [to be completed in a maximum of 4 weeks prior to randomisation (Visit 2)].
2. **Baseline, Study medication** - baseline assessments and randomisation to one of 2 treatment groups or matching placebo, will occur at Visit 2. At randomisation, patients will be stratified according to statin use and gender. The study medication period is 14 (+/-4) days. Patients will attend one outpatient visit during the 2-week study medication period (Visit 3) and will be telephoned on treatment day 14 if they are to take study medication for longer than 14 days.
3. **Follow-up** - the end of the study medication period will coincide with planned carotid endarterectomy. The last dose of study medication will be taken once a patient has been admitted to hospital during the day prior to surgery, for the purposes of PK and PD sampling (Visit 4). Carotid endarterectomy will take place the next day (Visit 5) according to local institution surgical guidelines. Final PK/PD samples will be taken during the day following surgery (Visit 6). A follow-up visit will occur 2 weeks after the end of study medication (Visit 7).

5. STUDY POPULATION

5.1. Number of Patients

For 2.5% significance for each comparison to placebo, overall 5% significance, 90% power, a difference between groups equivalent to 50% and between-subject standard deviation on the log scale of 0.7, a sample size of 27 modified intent-to-treat patients is needed per group. Allowing for a 20% rate of dropout or unevaluable plaque 33 patients per group are required, a total of 99 patients.

5.2. Eligibility Criteria

5.2.1. Inclusion Criteria

1. Male or female, >35 years of age.
2. Females of childbearing potential (i.e., who are not surgically sterile or postmenopausal with no menstrual period for a minimum of 6 months) during the period of administration of study medication (V2) until completion of follow-up procedures (V7); must be using one of the following contraceptive measures: abstinence OR one of the following methods: documented tubal ligation, documented placement of an intrauterine device (IUD), condom and diaphragm with spermicidal foam/gel/film/cream/suppository, condom with spermicidal foam/film/suppository. Postmenopausal women currently receiving hormone replacement therapy are eligible.
3. Male patients must be willing to abstain from sexual intercourse or use a condom and another form of contraception (eg. IUD, birth control pills taken by a female partner, diaphragm with spermicide) if engaging in sexual intercourse with a women who

could become pregnant between the administration of study medication until completion of the follow-up procedures.

4. Planned carotid endarterectomy within a timeframe compatible with recruitment for the study and able to comply with the requirements of the study, as deemed by the investigator.
5. Written, informed consent to participate.

5.2.2. Exclusion Criteria

A patient will not be eligible for inclusion in this study if any of the following criteria apply:

1. Recent myocardial infarction (within the previous 4 weeks).
2. Currently taking corticosteroids, warfarin, digoxin or a potent CYP3A4 inhibitor (see Section 8.2 Prohibited Medications for details).
3. Recent (<3 months) or ongoing acute infection or significant trauma associated with bruising and/or taking antibiotics. Prophylactic antibiotics for surgery are allowed.
4. Commencement or a change in the dose of a lipid-lowering therapy during the previous 4 weeks from randomisation
5. History of chronic liver disease (e.g. cirrhosis, hepatitis) OR ALT OR AST ≥1.5 times the upper limit of normal (ULN) at screening.
6. Diagnosis of systemic lupus erythematosus (SLE) or rheumatoid arthritis (RA).
7. Clinically significant anaemia (defined as haemoglobin <11g/dL in males, 10mg/dL in females).
8. History of severe renal impairment (serum creatinine >1.8mg/dL).
9. Unstable angina (Appendix 3).
10. History of asthma (defined as: episode of asthma in the previous 6 months or taking regular anti-asthmatic medication), anaphylaxis or anaphylactoid reactions, severe allergic responses.
11. Abuse of alcohol or drugs within the last 6 months.
12. Any factor or clinical disease state that, in the investigator's opinion, would preclude completion of a safe surgical procedure and/or completion of the study.
13. Use of an investigational drugs within 30 days or 5 half-lives of their last dose prior to starting the study, whichever is the longest.

5.2.3. Other Eligibility Criteria Considerations

To assess any potential impact on patient eligibility with regard to safety, the investigator must refer to the following document for detailed information regarding warnings, precautions, contraindications, adverse events, and other significant data pertaining to the investigational product being used in this study: Investigator Brochure for SB-480848.

5.2.4. Pharmacogenetics

Any patient who has given informed consent to participate in the clinical study, has met all the criteria required for entry into the clinical study and receives investigational product may take part in the pharmacogenetic study.

For Patients choosing to participate in the pharmacogenetic study, informed consent must be obtained prior to any blood being taken for this purpose. The informed consent for the PG research must be obtained in addition to the subject's consent to participate in the clinical study. Participation in the PG study is voluntary and refusal to participate will not indicate withdrawal from the clinical study. Refusal to participate will involve no penalty or loss of benefits to which the subject would otherwise be entitled.

No administration of investigational product beyond that detailed in the clinical protocol is associated with the pharmacogenetic study.

6. STUDY ASSESSMENTS AND PROCEDURES

A Time and Events Table is shown in Appendix 1.

Screening procedures should be conducted over a maximum period of 4 weeks prior to Visit 2 (baseline/randomisation).

The visit window for weekly visits is +/- 2 days. Study medication should be taken for 14(+/- 4) days. Visit 5 (day of surgery) must be conducted on the day following the last dose of study medication. Visit 6 must be conducted on the day after surgery, ie. 2 days following the last dose of study medication.

Visit 7 (follow-up) should be conducted 14 days (+/- 3 days) following the last dose of study medication.

6.1. Demographic and Baseline Assessments

The investigator, or designate, will collect the following data and perform the following procedures for all patients. Informed consent must be obtained from a patient prior to any study-specific procedures taking place.

6.1.1. Demography

The patient's date of birth, gender and race will be recorded in the CRF.

6.1.2. Medical history/medication

Relevant medical history (including, but not limited to, reason for carotid endarterectomy and degree of carotid stenosis, cardiovascular events, diabetes) and current medication being taken by the patient, will be recorded in the CRF. Smoking history will also be recorded.

6.1.3. Physical examination

The patient's height and weight will be recorded.

6.2. Safety

6.2.1. Vital signs

Blood pressure must be measured by the plethysmographic method (with a mercury column sphygmomanometer where available). All measurements will be made on the patient's non-dominant arm supported at heart level, using the same cuff size and the same equipment. If the patient's arm circumference is ≥32cm, a large blood pressure cuff should be used. Diastolic blood pressure will be measured at the disappearance of Korotkoff sounds (Phase V). If possible, measurements will be taken by the same staff member and using the same equipment throughout the study.

Blood pressure and heart rate will be measured as follows:

After the patient has sat quietly for at least 5 minutes, blood pressure and heart rate will be measured once. The measurements for sitting heart rate, systolic and diastolic blood pressure will be recorded in the CRF. If the patient is a smoker or uses any tobacco products, a period of 30 minutes without tobacco should be allowed before taking these measurements.

6.2.2. Electrocardiogram (ECG)

A standard 12-lead ECG will be recorded at Visits 2 (or at visit 1 if it is logistically easier) and 5. At Visit 5 the ECG can be taken up to 24 hours prior to surgery. If possible, the same equipment should be used throughout the study. A clear copy of the trace must be clearly labelled with patient number, protocol number, centre number and date, it must be signed and dated by the investigator and placed in the CRF. Any clinically significant ECG abnormalities at baseline should be documented in the baseline signs and symptoms section in the CRF. Additional clinically significant ECG abnormalities occurring during the study should be documented on the adverse event forms. ECGs will be reviewed by a core laboratory at the end of the study.

6.2.3. Chest X-ray

A chest X-ray will be performed at Visit 1 if one has not been done within the previous 6 months. Any clinically significant abnormalities will be recorded in the Medical History section of the CRF.

6.2.4. Clinical measurements

6.2.4.1. Haemoglobin A1c, Insulin

Fasting blood samples will be collected at Visit 2 for determination of glycated haemoglobin and insulin.

6.2.4.2. Peripheral Lymphocytes

Blood samples will be collected at Visits 2 and 7 for a microscopic examination of peripheral lymphocytes. Samples will be transferred to Quest Diagnostics and forwarded to a suitably qualified central laboratory. Sample handling and shipping instructions will be provided separately.

6.2.4.3. Haematology and Clinical Chemistry

Blood samples will be taken at each visit EXCEPT 4 and 6 (as indicated in Table 1) for routine haematology and clinical chemistry testing. All samples will be transferred to a central laboratory (Quest Diagnostics) for analyses. Sample handling and shipping instructions will be provided separately.

Parameters to be tested are listed below.

Biochemistry

Total Protein	LDH	Uric Acid	Potassium
Albumin	Total Bilirubin	Calcium	Chloride
ALT (SGPT)	Urea Nitrogen	Phosphorus	Bicarbonate
AST (SGOT)	Creatinine	Sodium	Alkaline Phosphatase
GGT			

Haematology

Platelet Count	*RBC Indices*:	WBC Count
RBC Count	MCV	*Automated WBC Differential:*
Haemoglobin	MCH	Neutrophils
Haematocrit	MCHC	Lymphocytes
		Monocytes
		Eosinophils
		Basophils

6.2.5. Pregnancy

Women of child-bearing potential should be taking adequate contraceptive measures (Section 5.2.1). However, in the unlikely event of pregnancy occurring during dosing with study medication or the follow-up period, details must be documented and reported to GSK. In addition any pregnancies brought to the attention of the investigator after this

period, and where it is known that study medication was taken at the time of conception, must also be reported.

Any pregnancies identified during the screening phase/prior to study medication administration do not need to be collected.

6.2.5.1. Pregnancy Testing

A urinary β-HCG pregnancy test will be performed for females of child-bearing potential at each visit during the study. If positive, a blood sample will be collected and sent to Quest Diagnostics who will perform a serum β-HCG test as confirmation.

6.2.5.2. Time Period for Collecting Pregnancy Information

The time period for collecting information on the occurrence of pregnancy will be identical to the time period for collecting AEs and SAEs as described in Section 10. However, pregnancies identified during the screening phase and prior to study medication administration do not need to be collected.

6.2.5.3. Action to be Taken if Pregnancy Occurs

Any female patient who becomes pregnant during the course of the study will be discontinued from study medication. The investigator, or his/her designee, will collect pregnancy information on the patient. The investigator, or his/her designee, will record pregnancy information on the appropriate form and submit it to GSK within 2 weeks of learning of a patient's pregnancy. The patient will also be followed to determine the outcome of the pregnancy. Information on the status of the mother and child will be forwarded to GSK. Generally, follow-up will be no longer than 6 to 8 weeks following the estimated delivery date. Any premature termination of the pregnancy will be reported.

While pregnancy itself is not considered to be an AE or SAE, any pregnancy complication or elective termination of a pregnancy for medical reasons will be recorded as an AE or a SAE, as described in Section 10.6, "Recording of AEs and SAEs" and will be followed as described in Section 10.8, "Follow-up of AEs and SAEs."

A spontaneous abortion is always considered to be a SAE and will be reported as described in Section 10, “Adverse Events (AE) and Serious Adverse Events (SAE).” Furthermore, any SAE occurring as a result of a post-study pregnancy and is considered reasonably related to the investigational product by the investigator, will be reported to GSK as described in Section 10.11, "Post-study AEs and SAEs." While the investigator is not obligated to actively seek this information in former study participants, he/she may learn of an SAE through spontaneous reporting.

6.2.6. Pharmacogenetic sampling

A whole blood sample (10ml) will be collected (in addition to any blood samples to be taken for the clinical study). Site staff must add the subject number to the specimen tube

(if a pre-printed label does not have the subject number). The blood sample will be taken on a single occasion (unless a duplicate sample is required due to inability to utilise the original sample). It is recommended that the blood sample be taken at the first available opportunity after randomisation (V2) but may be taken at any time during the study after randomisation.

6.3. Efficacy

Sample handling and shipping instructions will be provided separately.

6.3.1. Primary Efficacy

6.3.1.1. Plaque Lp-PLA2 Activity

The study medication dosing period is 14(+/- 4) days. The end of the study medication dosing period will coincide with the planned carotid endarterectomy for each patient according to local institution surgical guidelines. A patient will take their last dose of study medication the day before carotid endarterectomy.

Carotid endarterectomy is not a study-specific procedure and will be performed by a suitably qualified surgeon according to local practice.

The investigator, or designate, will be adequately trained on how to handle the excised plaque correctly. The collected plaque will be rinsed in saline solution, to remove blood traces and clots. The plaque will then be immediately frozen in liquid nitrogen and sent to Quest Diagnostics using dry ice transport. Quest Diagnostics will store the frozen samples and ship to GSK laboratories for analysis of Lp-PLA_2 activity. Full details of the plaque handling and transport procedure will be provided separately.

6.3.2. Secondary

6.3.2.1. Plasma Lp-PLA2 activity

A blood sample will be collected by the investigator, or designate, at Visits 2, and at V4 (or V3 as described in Table 1), V5, V6,V7 for measurement of Lp-PLA_2 activity.

- Fasting blood samples for Lp-PLA_2 are drawn at V2, V4 (or V3 pre-dose only), V5 and V7
- Non fasting blood samples Lp-PLA_2 are drawn at V6 and V4 (V3) post dose only.

Separated plasma will be shipped to a central laboratory (Quest Diagnostics) for storage. Analysis will take place at GSK laboratories.

6.3.2.2. Plasma and plaque Lp-PLA2 mass

Blood samples collected at Visits 2, 4, (or V3, as described in Table 1) 5, 6, 7, and extracted carotid plaque samples will be sent to Quest Diagnostics and then onto a GSK laboratory for the analyis for Lp-PLA_2 mass at a central laboratory (GSK).

6.4. Biomarker(s)

At Visits 2, 3, 5 and 7, the investigator, or designate, will collect and handle fasting blood samples for: Total cholesterol; high density lipoprotein (HDL); low density lipoprotein (LDL); triglycerides; high sensitivity C-reactive protein (hsCRP); CD40 ligand (CD40L); soluble intracellular adhesion molecules (sICAM); E-selectin; plasminogen activating inhibitor type 1 (PAI-1) activity and antigen; matrix metalloproteinase-9 (MMP-9). In addition, at visit 5, oxidised lipids,their metabolites including lyso-PC will be included in the analysis.

All samples will be analysed by suitably qualified central laboratories. The plaque oxidised lipids and their metabolites including lyso-PC will be analysed by an independent suitably qualified laboratory.

- Plaque samples removed during carotid endarterectomy will be analysed for concentrations/expression of the following biomarkers at a central laboratory (GSK): CD68 (Macrophages); CD3 (T cells); CD20 or 22 (B cells); α-actin (smooth muscle cells); CD40L; MMP -2 and -9; PAI –1; ICAM; IL-6; Lp-PLA_2 (mRNA expression), β-actin, GAPDH (Glyceraldehyde 3-Phosphate dehydrogenase) Axor 18 (a 7 Transmembrane Receptor), and Cyclophilin, oxidised lipids and their metabolites including lyso-PC.

In the event that there is insufficient plaque material to be able to carry the above analysis, lyso-PC will be omitted from the analysis.

Sample handling and shipping instructions will be provided separately.

6.5. Pharmacokinetics and Pharmcodynamics

6.5.1. PK/PD Sampling Schedule

All patients will participate in the PK/PD pharmacokinetic sampling. Blood samples for analysis will be collected as described in Table 1.

Table 1 PK/PD Sampling Schedule

Sampling Times	Rationale	Visit 4[1] Day prior to surgery (after admission)	Visit 6 Day after surgery
Just before dose	Trough Conc.	X	—
0.5-2 h after dose	Absorption Phase	X	—
2.5-4 h after dose	Peak Conc.	X	—
6-12 h after dose	Distribution Phase	X	—
Any time prior to discharge	Elimination Phase	—	X

1. if hospital practice does not allow PK/PD sampling to occur at the specifiied timepoints during Visit 4, where possible the sampling should be incorporated into the previous visit (3) when study medication is taken in clinic.

Visit 4 can be conducted by telephone, provided PK sampling has been carried out at V3. Patients not attending the Hospital for V4 will be telephoned to confirm that the subject will take their last dose of study medication with food in the morning of visit 4 (the day immediately prior to surgery). The patient should be reminded that Visit 5 is a fasting visit and the patient must not eat or drink with the exception of water, after 11pm on visit 4.

Patients attending the Hospital for visit 4, will be instructed not to take their dose of study medication until after admission to hospital.

The first PK and PD (Lp-PLA2) blood sample at Visit 4 will be collected just before taking study medication. The time of dosing and sample collection will be recorded in the CRF and samples taken within the following time windows:

- 0.5 - 2 hours post dose
- 2.5 - 4 hours post dose
- 6 - 12 hours post dose

If it is not logistically possible to take the PK/PD (Lp-PLA2) samples during Visit 4 (0.5 - 2, 2.5 - 4; and 6 - 12 hours post dose) because the patient is unable to arrive early in the Hospital, then these samples can be taken at Visit 3.

The final PK blood samples will be taken at any time on the day after surgery

Lp-PLA2 Blood sampling: A single blood sample will be collected at V2, V5, V6 and V7. At Visit 4 (or V3 if it is not logistically possible to take these samples at V4*)* blood samples will be collected as described in Table 1 (Pre-dose, 0.5 –2hr, 2.5 –4hr and 6 - 12hr).

Every effort should be made to collect samples at times spread throughout the time window (i.e., avoid collections from all patients at the same time within a window or only at the extremes of a time window).

In addition to PK/PD samples, another blood sample will be taken at each timepoint. At the end of the study, if appropriate, these samples may be used to measure levels of some of the defined biomarker(s) (Section 6.4) which have demonstrated a change throughout the course of the study.

Samples will be prepared and shipped to Quest Diagnostics. Full details will be provided separately. Quest Diagnostics will store and ship the samples to GSK, where they will be analysed using approved assay techniques.

7. INVESTIGATIONAL PRODUCT(S)

7.1. Description of Investigational Product

SB-480848 will be provided by GSK as a pink, film coated tablet containing 20mg of SB-480848. Matching placebo tablets, indistinguishable in appearance from the SB-480848 tablets, will also be provided by GSK.

7.2. Dosage and Administration

At Visit 2, patients will be randomised to one of three dose groups:

- SB-480848 40mg
- SB-480848 80mg
- Matching placebo

Patients will be provided with 2 bottles and will take 2 tablets from each bottle per day. Patients will continue with their allocated dose group for the 14+/-4 day treatment period.

Study medication should be taken in the morning with food. If PK/PD assessments are to be made during Visit 4 (Day Prior to Surgery) patients should delay taking study medication until instructed to do so after admission to hospital.

On fasting visit days, morning doses of study medication will be taken in the clinic after all fasting laboratory specimens have been obtained.

7.3. Dose Rationale

PK/PD modelling has been undertaken to assess the dose/drug concentration/plasma Lp-PLA_2 enzyme activity inhibition relationship for SB-480848. Modelling and simulation suggest that peak and trough inhibition of plasma Lp-PLA_2 enzyme activity appear to begin to plateau at doses around 80 mg. It is anticipated that once daily doses of 80 and 40 mg SB-480848 are likely to result in approximately 85-90% and 60-65% trough (24-hour post-dose) plasma Lp-PLA_2 enzyme inhibition at steady state, respectively. The

level of correlation between plaque and plasma enzyme inhibition is not known, however, this will be investigated in the study.

7.3.1. Pharmacogenetics

Pharmacogenetics (PG) is a study of variability in drug response due to hereditary factors in different populations. There is increasing evidence that an individual's genetic composition (ie. his or her genotype) may impact on drug response or clinical outcome

The aim is to look at the impact of genetic variation on response of SB-480848. This may include trying to identify any genetic markers (variation in a number of genes) that could predict the outcome of investigational treatment response (safety and/or efficacy).

This genetic research is not designed to determine whether other members of the patient's family are at risk of developing atherosclerosis. Neither is the research designed to generate information relevant to the patient's decision regarding whether or not to have children. Participation in the pharmacogenetic portion of the protocol is voluntary and is independent of participation in the core protocol.

7.4. Blinding

SB-480848 and matching placebo will be provided for blinding purposes as tablets that will be indistinguishable in appearance. Neither the patient nor the investigator will know which study medication the subject is receiving.

Individual code-breaks will be provided to the investigator. Each code-break will identify the container number and will include an unblinding panel which can be removed by scratching with a coin to reveal the container contents.

Only in the case of an emergency, when knowledge of the investigational product is essential for the clinical management or welfare of the patient, the investigator may unblind a patient’s treatment assignment. If the blind is broken for any reason, the investigator must notify GSK **immediately** of the unblinding incident without revealing the patient’s study treatment assignment. In addition, the investigator will record the date and reason for revealing the blinded treatment assignment for that patient in the appropriate CRF.

If a serious adverse event (SAE; as defined in Section 10.2, "Definition of a SAE") is reported to GSK, Global Clinical Safety and Pharmacovigilance (GCSP) staff may unblind the treatment assignment for the individual patient. If an expedited regulatory report to one or more regulatory agencies is required, the report will identify the patient’s treatment assignment. When applicable, a copy of the regulatory report may be sent to investigators in accordance with relevant regulations, GSK policy, or both.

7.5. Treatment Assignment

Patients will be assigned to study treatment in accordance with the randomisation schedule. The randomisation schedule will be generated by the GSK statistician.

The Registration and Medication Ordering system (RAMOS) is a telephone based system which will be used to register patients, randomise patients, and allocate double blind medication bottles. Randomisation will be stratified according to statin use and gender.

Please note that the order of drug dispensing will not always be in sequential order.

Note: This telephone call is very important since it will be the only mechanism of identifying which study medication will be assigned to a patient. In addition, this system tracks all shipments of study medication and will also be responsible for resupplies.

The following calls must be made by the investigator (or designee) to RAMOS to perform the following functions:

Visit One	Registration for study SB-480848/010
Visit Two	Randomisation to one of three treatment arms and allocation of appropriate bottle numbers.<br><br>At randomisation, patients will be stratified according to statin use and gender.
As necessary	Notification of withdrawal from the study

All calls to RAMOS are confirmed with faxes which are sent to the investigator upon completion of each call. Detailed RAMOS user instructions and worksheets will be provided at study start. All RAMOS faxes and worksheets must be retained in the Investigator's study file.

7.6. Packaging and Labeling

SB-480848 or matching placebo tablets will be supplied in bottles containing tablets of 480848 20mg or placebo to match for 18 days plus 2 days overage.

The container number on the study medication treatment pack will also be recorded in the CRF.

The contents of the label will be in accordance with all applicable regulatory requirements.

Labels for study medication will be printed in the local language. Details included on the labels are as follows:

- Container number
- Quantity statement
- Protocol number
- Dosing instructions

- Storage conditions
- "Keep out of reach of children"
- "For Clinical Trial Use only"
- Company Address
- Expiry Date

7.7. Preparation

No additional preparation required.

7.8. Handling and Storage

Investigational product must be dispensed or administered according to procedures described herein. Only patients enrolled in the study may receive investigational product, in accordance with all applicable regulatory requirements. Only authorized site staff may supply or administer investigational product. All investigational products must be stored in a secure area with access limited to the investigator and authorized site staff and under physical conditions that are consistent with investigational product-specific requirements.

Prior to dispensing, all study medication will be kept safely locked in a dry place at controlled room temperature (20-25°C).

7.9. Product Accountability

The investigator is responsible for investigational product accountability, reconciliation, and record maintenance. In accordance with all applicable regulatory requirements, the investigator or designated site staff must maintain investigational product accountability records throughout the course of the study. This person(s) will document the amount of investigational product received from GSK, the amount supplied and/or administered to and returned by patients, if applicable.

A log for recording receipt of and use of study medications will be maintained by the designated personnel at the Investigator site.

7.10. Assessment of Compliance

The number of tablets dispensed at Visit 2 as well as the calculated percent compliance for study medication will be recorded in the CRF. The percentage compliance may be read from a table provided in the CRF. The following calculation has been used:

$$\frac{\{\text{No. dispensed - No. returned}\}}{\text{No. of tablets to be taken per day} \times \text{No. days since the last visit}} \times 100$$

The investigator will not be required to complete the above equation but will still be required to record whether compliance was ≥80% and ≤120%.

Patients will be instructed to return any unused study medication at the end of the treatment period.

7.11. Treatment of Investigational Product Overdose

Any signs or symptoms of overdosage will be treated symptomatically. No specific antidote is known.

7.12. Occupational Safety

Investigational product is not expected to pose significant occupational safety risk to site staff under normal conditions of use and administration. A Material Safety Data Sheet (MSDS) describing occupational hazards and recommended handling precautions either will be provided to the investigator, where this is required by local laws, or is available upon request from GSK.

8. CONCOMITANT MEDICATIONS AND NON-DRUG THERAPIES

8.1. Permitted Medications

All concomitant medications taken during the study will be recorded in the CRF with indication, dose information, and dates of administration. During the planned Carotid endarterectomy (visit 5) drugs administered for anaesthesia will be recorded in the CRF with the total dose administered and the date of administration (visit 5 date).

Commencement or a change in a lipid lowering medication is not permitted during the study (V1 to V7) or during the 4 weeks prior to randomisation.

8.2. Prohibited Medications

Treatment with corticosteroids, warfarin and/or digoxin is prohibited during the study. In addition, the following potent CYP3A4 inhibitors are prohibited:

HIV Antivirals	Potent Macrolides	Azole Antifungals
Delaviridine	Clarithromycin	Fluconazole
Indinavir	Erythromycin	Itraconazole
Nelfinavir	Troleandomycin	Ketoconazole
Ritonavir		Voriconazole
Saquinavir		

9. PATIENT COMPLETION AND WITHDRAWAL

9.1. Patient Completion

All patients will be considered to have completed the study upon completion of all the assessments and procedures up to and including Visit 7.

Any patient who enters the study, (i.e. gives written informed consent) but does not, **for any reason** complete the study according to the definitions given above, will be considered a withdrawal, irrespective of whether they have received any study medication.

9.2. Patient Withdrawal

9.2.1. Patient Withdrawal from Study

Each patient participating in the study will be assigned a unique CRF/patient number. Enrolment to the study will continue until 99 patients have been randomised into the study. Study medication given to withdrawn patients will **not** be reused.

Patients who terminate their participation in the study due to adverse experiences should be followed up as appropriate (see Section 10) in order to determine the final outcome.

Every effort should be made to follow up patients who withdraw or are withdrawn from the study prematurely. CRFs of **all** patients entered into the study must be completed. For patients who withdraw prior to randomisation (i.e. during the run-in period), complete the Study Continuation/Withdrawal page in the CRF.

For patients withdrawing after randomisation complete the Study Conclusion page and the Early Withdrawal (7) Visit. Reasons for withdrawal must be noted on the Study Conclusion page for all randomised patients who do not complete the study. At the patient's final visit, collect any unused study medication, and record the date of the last dose of study medication on the Study Conclusion page.

9.2.2. Patient Withdrawal from Investigational Product

Every effort must be made by the Investigator to keep patients in the study. However, a patient may be discontinued prior to completion of the study for the following reasons:

- Adverse experience (see Section 10).
- Protocol Violation: including lack of compliance with visit scheduling, or treatment with prohibited concomitant medications.
- Patient lost to follow-up.
- The patient requests an early discontinuation (**not** due to an adverse experience).
- Termination of the study by GSK

- Other: Document reason in the CRF.

All adverse experiences leading to withdrawal of a patient must be fully documented and followed-up as appropriate (see Section 10). To ensure that all withdrawals due to adverse experiences are correctly identified, "adverse experience" should only be checked as the reason for withdrawal on the Study Conclusion page for those patients for whom an adverse experience was considered to be the direct cause of the patient withdrawing from the study. This is particularly important when adverse experiences are ongoing at the time of withdrawal, but the reason for withdrawal is not related to the adverse experiences.

9.3. Screen and Baseline Failures

Data will not be collected on the database for screen and/or baseline failures i.e. patients who withdraw from the study following informed consent, but before receiving double-blind study medication.

10. ADVERSE EVENTS (AE) AND SERIOUS ADVERSE EVENTS (SAE)

The investigator is responsible for the detection and documentation of events meeting the criteria and definition of an AE or SAE as provided in this protocol. During the study, when there is a safety evaluation, the investigator or site staff will be responsible for detecting AEs and SAEs, as detailed in this section of the protocol.

10.1. Definition of an AE

Any untoward medical occurrence in a patient or clinical investigation subject, temporally associated with the use of a medicinal product, whether or not considered related to the medicinal product.

An AE can therefore be any unfavourable and unintended sign (including an abnormal laboratory finding), symptom, or disease (new or exacerbated) temporally associated with the use of a medicinal product. For marketed medicinal products, this also includes failure to produce expected benefits (i.e. lack of efficacy), abuse or misuse.

Examples of an AE **includes**:

- Significant or unexpected worsening or exacerbation of the condition/indication under study. See Section 10.3, “Lack of Efficacy”, for additional information.
- Exacerbation of a chronic or intermittent pre-existing condition including either an increase in frequency and/or intensity of the condition.
- New conditions detected or diagnosed after investigational product administration even though it may have been present prior to the start of the study.
- Signs, symptoms, or the clinical sequelae of a suspected interaction.

- Signs, symptoms, or the clinical sequelae of a suspected overdose of either investigational product or a concurrent medication (overdose per se should not be reported as an AE/SAE).
- Significant failure of expected pharmacological or biological action. See Section 10.3, “Lack of Efficacy” for additional information.

Examples of an AE **does not include** a/an:

- Medical or surgical procedure (e.g., endoscopy, appendectomy); the condition that leads to the procedure is an AE.
- Situations where an untoward medical occurrence did not occur (social and/or convenience admission to a hospital).
- Anticipated day-to-day fluctuations of pre-existing disease(s) or condition(s) present or detected at the start of the study that do not worsen.
- The disease/disorder being studied, or expected progression, signs, or symptoms of the disease/disorder being studied, unless more severe than expected for the patient’s condition.

For GSK clinical studies, AEs may include pre- or post-treatment events that occur as a result of protocol-mandated procedures (i.e., invasive procedures, modification of patient’s previous therapeutic regimen). Planned carotid endarterectomy is not a protocol-mandated procedure.

10.2. Definition of a SAE

A serious adverse event is any untoward medical occurrence that, at any dose:

a results in death.

b is life-threatening.

NOTE: The term 'life-threatening' in the definition of 'serious' refers to an event in which the patient was at risk of death at the time of the event. It does not refer to an event, which hypothetically might have caused death, if it were more severe.

c requires hospitalisation or prolongation of existing hospitalisation.

NOTE: In general, hospitalisation signifies that the patient has been detained (usually involving at least an overnight stay) at the hospital or emergency ward for observation and/or treatment that would not have been appropriate in the physician’s office or out-patient setting. Complications that occur during hospitalisation are AEs. If a complication prolongs hospitalisation or fulfills any other serious criteria, the event is serious. When in doubt as to whether “hospitalisation” occurred or was necessary, the AE should be considered serious.

Hospitalisation for elective treatment of a pre-existing condition that did not worsen from baseline is not considered an AE.

d results in disability/incapacity, or

NOTE: The term disability means a substantial disruption of a person's ability to conduct normal life functions. This definition is not intended to include experiences of relatively minor medical significance such as uncomplicated headache, nausea, vomiting, diarrhoea, influenza, and accidental trauma (e.g. sprained ankle) which may interfere or prevent everyday life functions but do not constitute a substantial disruption.

e is a congenital anomaly/birth defect.

f Medical or scientific judgement should be exercised in deciding whether reporting is appropriate in other situations, such as important medical events that may not be immediately life-threatening or result in death or hospitalisation but may jeopardize the patient or may require medical or surgical intervention to prevent one of the other outcomes listed in the above definition. These should also be considered serious. Examples of such events are invasive or malignant cancers, intensive treatment in an emergency room or at home for allergic bronchospasm, blood dyscrasias or convulsions that do not result in hospitalisation, or development of drug dependency or drug abuse.

10.2.1. Surgery-Related Events or Outcomes Not Qualifying as SAEs

Carotid endarterectomy is associated with significant morbidity/mortality (risk of stroke, bleeding, death). Such post-operative events will not be recorded as SAEs.

10.3. Lack of Efficacy

"Lack of efficacy" per se will not be reported as an AE. The signs and symptoms or clinical sequelae resulting from lack of efficacy will be reported if they fulfil the AE or SAE definition (including clarifications).

10.4. Clinical Laboratory Abnormalities and Other Abnormal Assessments as AEs and SAEs

Abnormal laboratory findings (e.g., clinical chemistry, hematology, urinalysis) or other abnormal assessments (e.g., ECGs, chest x-ray) that are judged by the investigator as **clinically significant** will be recorded as AEs or SAEs if they meet the definition of an AE, as defined in Section 10.1 ("Definition of an AE"), or SAE, as defined in Section 10.2 ("Definition of a SAE"). Clinically significant abnormal laboratory findings or other abnormal assessments that are detected during the study or are present at baseline and significantly worsen following the start of the study will be reported as AEs or SAEs. However, clinically significant abnormal laboratory findings or other abnormal assessments that are associated with the disease being studied, unless judged by the investigator as more severe than expected for the patient's condition, or that are present or detected at the start of the study and do not worsen, will **not** be reported as AEs or SAEs.

The investigator will exercise his or her medical and scientific judgement in deciding whether an abnormal laboratory finding or other abnormal assessment is clinically significant.

10.5. Time Period, Frequency, and Method of Detecting AEs and SAEs

Any pre-existing conditions or signs and/or symptoms present in a patient prior to the start of the study (i.e. before informed consent) should be recorded in the Medical/Surgical History section within the CRF.

In order to fulfil international reporting obligations, SAEs that are related to study participation (e.g. procedures, invasive tests, change from existing therapy) or are related to a concomitant medication will be collected and recorded from the time the patient consents to participate in the study until he/she is withdrawn or completes.

Any medical occurrences which occur after informed consent is obtained but prior to starting active or randomised treatment will be documented on the Baseline Signs & Symptoms section within the CRF.

All AEs occurring after administration of the first dose of study medication and on or before the final visit must be reported on the Adverse Event form in the CRF. All AEs must be recorded irrespective of whether they are considered drug related.

At each visit/assessment in the period defined above, AEs will be evaluated by the investigator and recorded in the appropriate AE/Serious AE section of the CRF.

Any AEs already documented at a previous assessment and designated as ongoing, should be reviewed at subsequent visits as necessary. If these have resolved, the documentation in the CRF should be completed. If an AE changes in intensity/frequency then this should be recorded as a separate event (i.e. a new record started).

As a consistent method of soliciting AEs, the patient should be asked a non-leading question such as:

"Have you felt different in any way since starting the new treatment/or since the last visit?"

or

"How do you feel?"

10.6. Recording of AEs and SAEs

When an AE/SAE occurs, it is the responsibility of the investigator to review all documentation (e.g., hospital progress notes, laboratory, and diagnostics reports) relative to the event. The investigator will then record all relevant information regarding an AE/SAE on the CRF. It is not acceptable for the investigator to send photocopies of the

patient's medical records to GSK in lieu of completion of the appropriate AE/SAE CRF pages. However, there may be instances when copies of medical records for certain cases are requested by GSK. In this instance, all patient identifiers will be blinded on the copies of the medical records prior to submission to GSK.

The investigator will attempt to establish a diagnosis of the event based on signs, symptoms, and/or other clinical information. In such cases, the diagnosis should be documented as the AE/SAE and not the individual signs/symptoms.

10.7. Evaluating AEs and SAEs

10.7.1. Assessment of Intensity

The investigator will make an assessment of intensity for each AE and SAE reported during the study. The assessment will be based on the investigator's clinical judgement. The intensity of each AE and SAE recorded in the CRF should be assigned to one of the following categories:

Mild: An event that is easily tolerated by the patient, causing minimal discomfort and not interfering with everyday activities.

Moderate: An event that is sufficiently discomforting to interfere with normal everyday activities.

Severe: An event that prevents normal everyday activities.

An AE that is assessed as severe should not be confused with a SAE. Severity is a category utilized for rating the intensity of an event; and both AEs and SAEs can be assessed as severe. An event is defined as 'serious' when it meets one of the pre-defined outcomes as described in Section 10.2, "Definition of a SAE".

10.7.2. Assessment of Causality

The investigator is obligated to assess the relationship between investigational product and the occurrence of each AE/SAE. The investigator will use clinical judgement to determine the relationship. Alternative causes, such as natural history of the underlying diseases, concomitant therapy, other risk factors, and the temporal relationship of the event to the investigational product will be considered and investigated. The investigator will also consult the CIB/IB and/or Product Information, for marketed products, in the determination of his/her assessment.

There may be situations when an SAE has occurred and the investigator has minimal information to include in the initial report to GSK. However, it is very important that the investigator always make an assessment of causality for every event prior to transmission of the SAE CRF to GSK. The investigator may change his/her opinion of causality in light of follow-up information, amending the SAE CRF accordingly. The causality assessment is one of the criteria used when determining regulatory reporting requirements.

The investigator will provide the assessment of causality as per instructions on the SAE form in the CRF.

10.8. Follow-up of AEs and SAEs

After the initial AE/SAE report, the investigator is required to proactively follow each patient and provide further information to GSK on the patient's condition.

All AEs and SAEs documented at a previous visit/contact and are designated as ongoing, will be reviewed at subsequent visits/contacts.

All AEs and SAEs will be followed until resolution, until the condition stabilizes, until the event is otherwise explained, or until the patient is lost to follow-up. Once resolved, the appropriate AE/SAE CRF page(s) will be updated. The investigator will ensure that follow-up includes any supplemental investigations as may be indicated to elucidate the nature and/or causality of the AE or SAE. This may include additional laboratory tests or investigations, histopathological examinations, or consultation with other health care professionals.

GSK may request that the investigator perform or arrange for the conduct of supplemental measurements and/or evaluations to elucidate as fully as possible the nature and/or causality of the AE or SAE. The investigator is obligated to assist. If a patient dies during participation in the study or during a recognized follow-up period, GSK will be provided with a copy of any post-mortem findings, including histopathology.

New or updated information will be recorded on the originally completed "SAE" CRF, with all changes signed and dated by the investigator. The updated SAE CRF should be resent to GSK within the time frames outlined in Section 10.9.

10.9. Prompt Reporting of SAEs to GSK

SAEs will be reported promptly to GSK as described in the following table once the investigator determines that the event meets the protocol definition of an SAE.

10.9.1. Timeframes for Submitting SAE Reports to GSK

	Initial SAE Reports		Follow-up Information on a Previously Reported SAE	
Type of SAE	Time Frame	Documents	Time Frame	Documents
All SAEs	24 hrs	"SAE" CRF pages	24 hrs	Updated "SAE" CRF pages

10.9.2. Completion and Transmission of the SAE Reports

Once an investigator becomes aware that an SAE has occurred in a study patient, she/he will report the information to GSK within 24 hours as outlined in Section 10.9. “Prompt Reporting of SAEs to GSK”. The SAE CRF will always be completed as thoroughly as possible with all available details of the event, signed by the investigator (or designee), and forwarded to GSK within the designated time frames. If the investigator does not have all information regarding an SAE, he/she will not wait to receive additional information before notifying GSK of the event and completing the form. The form will be updated when additional information is received.

The investigator will always provide an assessment of causality at the time of the initial report as described in Section 10.7.2 “Assessment of Causality”.

Facsimile transmission of the “SAE” CRF is the preferred method to transmit this information to the project contact for SAE receipt. In rare circumstances and in the absence of facsimile equipment, notification by telephone is acceptable, with a copy of the "SAE" CRF sent by overnight mail. Initial notification via the telephone does not replace the need for the investigator to complete and sign the SAE CRF within the time frames outlined in Section 10.9, “Prompt Reporting of SAEs to GSK”.

GSK will provide a list of project contacts for SAE receipt, fax numbers, telephone numbers, and mailing addresses.

The following pages of the CRF must accompany the SAE forms that are forwarded to GSK: “Demography”, “Medical History”, “Concomitant Medications”, “Study Medication Records”, and “Form D” (if applicable).

10.10. Regulatory Reporting Requirements for SAEs

The investigator will promptly report all SAEs to GSK in accordance with the procedures detailed in Section 10.9, "Prompt Reporting of SAEs to GSK." GSK has a legal responsibility to notify, as appropriate, both the local regulatory authority and other regulatory agencies about the safety of a product under clinical investigation. Prompt notification of SAEs by the investigator to the appropriate project contact for SAE receipt is essential so that legal obligations and ethical responsibilities towards the safety of other patients are met.

The investigator, or responsible person according to local requirements, will comply with the applicable local regulatory requirements related to the reporting of SAEs to regulatory authorities and the Institutional Review Board (IRB)/Independent Ethics Committee (IEC).

Expedited Investigator Safety Reports (EISR) are prepared according to GSK policy and are forwarded to investigators as necessary. An EISR is prepared for a SAE that is both attributable to investigational product and unexpected. The purpose of the EISR is to fulfil specific regulatory and Good Clinical Practice (GCP) requirements, regarding the product under investigation.

An investigator who receives an EISR describing a SAE or other specific safety information from GSK will file it with the Investigator Brochure and will notify the IRB or IEC, if appropriate according to local requirements.

10.11. Post-Study AEs and SAEs

A post-study AE/SAE is defined as any event that occurs outside of the AE/SAE detection period defined in Section 10.5, “Time Period, Frequency, and Method of Detecting AEs and SAEs”, of the protocol.

Investigators are not obligated to actively seek AEs or SAEs in former study participants. However, if the investigator learns of any SAE, including a death, at any time after a patient has been discharged from the study, and he/she considers the event reasonably related to the investigational product, the investigator will promptly notify GSK.

10.12. SAEs Related to Study Participation

An SAE considered related to study participation (e.g., procedures, invasive tests, a change in existing therapy), even if it occurs during the pre- or post-treatment period, will be reported promptly to GSK (see Section 10.9, "Prompt Reporting of SAEs to GSK").

11. DATA ANALYSIS AND STATISTICAL CONSIDERATIONS

11.1. Hypotheses

The primary objective is to show that SB-480848 shows superiority versus placebo in lowering carotid plaque Lp-PLA_2 activity levels. As carotid plaque can only be obtained following surgery the null hypothesis to be tested is that there is no difference in plaque Lp-PLA_2 activity levels between placebo and active groups following 14 (+/- 4) days of dosing. The alternative hypothesis is that there is a difference between placebo and one or more active dose levels. Testing will be two-sided.

11.2. Treatment Comparisons of Interest

11.2.1. Primary Comparisons of Interest

The primary objective of the study will be assessed by tests of hypotheses. The value of carotid plaque Lp-PLA_2 activity will be compared between placebo and each active group. The primary analysis will be performed on the modified intent-to-treat population (see Section 11.5 for definition of this population). A simple Bonferoni adjustment will be made to allow for the two active groups each being compared to placebo. This is a conservative approximation to two-sided Dunnett’s test for multiple comparison to control in a general linear model. [CHAPMAN, 1993] An overall alpha level of 5% will be used i.e. an alpha for each of the two comparisons to placebo of 2.5%.

11.2.2. Other Comparisons of Interest

The primary comparison described above will also be performed using the evaluable population (see Section 11.5). The analyses of carotid plaque Lp-PLA_2 activity will be repeated for carotid plaque Lp-PLA_2 mass. Details for all other formal comparisons of interest are given in Section 11.7.2 and Section 11.7.3.

11.3. Interim Analysis

None planned.

11.4. Sample Size Considerations

11.4.1. Sample Size Assumptions

A pilot study, SB 480848/021, is an experimental study in untreated patients scheduled for carotid endartarectomy surgery [TRELFA]. It uses the plaque collection technique proposed as for this study and will allow this to be optimised prior to initiation of this study. Excised plaques are being assayed for Lp-PLA_2 and biomarker levels to characterise plaque in untreated patients. This will help to put results from treated patients into context and to allow sample size calculation for this study. An interim analysis of 13 plaques showed plaque Lp-PLA_2 activity levels were skewed and a log-transformation was appropriate. The between-subject standard deviation on the log-transformed scale was 0.6225. The clinically relevant difference is a 50% inhibition in plaque Lp-PLA_2 activity.

The observed between-subject standard deviation is an estimate based on a small number of patients. Therefore for the base case this has been rounded up to 0.7. An inhibition of 50% corresponds to a difference between groups of 0.693 on the log scale or a relative effect (difference in means)/(between-subject standard deviation) of about 1. Across a whole range of studies an effect of this size is usually considered a large effect.

For 2.5% significance for each comparison to placebo, overall 5% significance, 90% power, a difference between groups equivalent to 50% and between-subject standard deviation on the log scale of 0.7, a sample size of 27 modified intent-to-treat patients is needed per group. Allowing for a 20% rate of dropout or unevaluable plaque 33 patients per group are required, a total of 99 patients.

11.4.2. Sample Size Sensitivity

Sample size sensitivity for this study is shown in Table 2 below based on 27 modified intent-to-treat patients and a range of clinically relevant inhibitions and between-subject standard deviations on the log-scale:

Table 2 Statistical power (%) for 27 patients per group

Between-subject standard deviation on log-scale	Clinically relevant level of inhibition		
	40%	50%	60%
0.6	79%	97%	>99%
0.7	65%	90%	99%
0.8	52%	81%	97%

For a clinically relevant inhibition of 50% and between-subject standard deviation on the log scale of 0.7 if there are 21 modified intent-to-treat patients the power is 81%, while if there are 24 modified intent-to-treat patients the power is 86%.

11.4.3. Sample Size Re-Estimation

No sample size re-estimation will be performed within this study. However if final results from study SB-480848/021 show significantly different variability from those seen at the interim analysis then the power and sample size of this study will be re-assessed.

11.5. Analysis Populations

The populations to be evaluated are:

- modified intent-to-treat: patients receiving at least one-dose of study medication and having valid Lp-PLA_2 activity data from carotid plaque.
- intent-to-treat: patients receiving at least one-dose of study medication and having at least one efficacy assessment post-randomisation
- evaluable: subset of modified intent-to-treat population of patients who have at least 10 days of study medication, are between 80 and 120% compliant with medication, as defined in Section 11.6.3, and have carotid endarterectomy surgery within 24 hours of last dose of study medication.
- safety: patients receiving at least one dose of study medication.

For efficacy, the modified intent-to-treat population is of primary interest. The primary analysis will be repeated in the evaluable patient population as a sensitivity check. If the analyses are consistent then all plaque efficacy analyses will be performed on the modified intent-to-treat population only. If the analyses are inconsistent then data from non-evaluable patients will be reviewed in detail to identify the reason for the discrepancy, if possible. Unless a reason for discrepancy is apparent other analyses will be repeated in both populations. For plasma efficacy analyses the dropout rate and possible relation of the dropout pattern to treatment group will be assessed. If appropriate, plasma efficacy analyses may be repeated for the intent-to-treat population.

11.5.1. Data Sets

The primary statistical analysis will use the modified intent–to-treat patient population using observed data. The primary timepoint of interest is the day of carotid endarterectomy surgery.

11.6. General Considerations for Data Analysis

11.6.1. Withdrawal

If a subject withdraws prior to carotid endarterectomy surgery they cannot be included in the primary analysis as they will have no plaque data available.

11.6.2. Missing Data

In this study plaque Lp-PLA$_2$ activity data will only be obtained at the end of treatment. The behaviour of this data and plaque biomarker data after two weeks of SB 480848 treatment is not known. Neither is the relationship to plasma data known. Therefore no imputation of plaque data will be performed.

In this study plasma samples will be taken during treatment, Visit 3. However this visit is before drug plasma levels are modelled to have achieved steady state. Therefore to keep plaque and plasma data on as consistent a basis as possible no imputation or carrying forward of plasma data will be performed.

If no Visit 2 (randomisation), value is available for an efficacy assessment variable then the baseline value will remain missing and the subject cannot be used in any summary of change from baseline for this variable. For laboratory safety assessments if no Visit 2, randomisation visit, value is available but a Visit 1, screening visit, value is available then this will be used as baseline.

11.6.3. Derived and Transformed Data

The SB-480848/021 plaque Lp-PLA$_2$ activity data indicates a log-transformation should be used [TRELFA]. The appropriateness of this transformation for post-treatment data to achieve standard analysis assumptions of normality and constant variance will be assessed and untransformed data used if more appropriate.

Percent inhibition of plasma Lp-PLA$_2$ levels will be calculated as 100*(baseline-value)/baseline, where baseline is the Visit 2, randomisation value, fasting value of Lp-PLA$_2$. Maximum inhibition is therefore 100%.

Percentage compliance will be calculated as 100*(tablets taken)/(days of dosing).

11.6.4. Assessment Windows

All data will be reported according to the nominal time point for which it was recorded.

11.6.5. Other Issues

Any deviations from the analyses described in the protocol will be described in the Reporting and Analysis Plan (RAP) and final Study Report as appropriate.

No separate analyses of subgroups will be performed, though summaries by risk factors will be produced. The effects of risk factors will be assessed by including them in an analysis of covariance.

11.7. Efficacy Analysis

11.7.1. Primary Analysis

It is not known where the doses might fall in terms of any dose response, though plasma results from preclinical and repeat dose study SB-480848/002 show a monotonic dose response [FINNERTY]. After analysis of variance, adjusting for the stratification by statin use and gender, each of the active groups will be compared to placebo.

The assumptions of normality and constant variance will be assessed graphically following a log-transformation. If the assumptions are not met because variability appears to be constant untransformed data will be used. If the assumptions are not met because normality is not appropriate a non-parametric analysis using the rank-sum test will be performed without adjustment for covariates due to the potential for small cell size. [CHAPMAN, 1996]

11.7.2. Secondary Analysis

Plasma Lp-PLA_2 levels will be analysed as longitudinal data, using mixed effects for repeat measures, and testing for treatment effect. The analysis will be performed using the multiple record per patient form for the data so patients with partial data are included in the analysis. Fixed effects to be included in the model are the stratification variables of statin use and gender, the known risk factor of age, and treatment, in the order given. Subject will be a random effect.

Initially time will be modelled as a linear effect. This will be assessed by plotting overlaid individual subject graphs. If linearity is not appropriate interaction of time and treatment will be investigated: a 10% significance level will be used to declare effects as formally significant.

If there is a higher than anticipated level of dropouts the analysis will be repeated in the intent-to-treat population to assess whether there is any differential effect due to treatment related dropout. Results of modelling of Watanabe rabbit plasma $LpPLA_2$ levels will be reviewed prior to unblinding of this study and the proposed analysis modified if appropriate.

11.7.3. Other Efficacy Analysis

The primary analysis will be repeated for change in plasma Lp-PLA_2 level from baseline, for change in plasma biomarkers, and for plaque biomarkers.

Analysis of the relation of plasma and plaque Lp-PLA_2 levels and other efficacy or biomarker data is primarily experimental. The pilot study SB 480848/021 is examining the relation between plasma and plaque Lp-PLA_2 levels in untreated patients [TRELFA]. Any such relation will form the starting point for the examination of relationship in this study, with any possible modification by dosing with SB-480848 of particular interest. Results from Watanabe rabbit studies will also be used to guide the exploration. Exploratory graphical analyses between biomarkers and Lp-PLA_2 in blood and plaque will be performed, including any possible differential effect of Lp-PLA_2 inhibition. If appropriate further formal analyses will be performed.

Dose response effect of SB-480848 on plaque and plasma Lp-PLA_2 levels will be assessed. A general linear model with effects for the stratification variables of statin use and gender, the known risk factor of age, baseline LpPLA_2 and dose level (0, 40, 80), in the order given. A linear dose contrast will be fitted. Interactions of treatment with explanatory variables will be assessed: a 10% significance level will be used to declare effects as formally significant. Baseline biomarker data will also be explored as covariates using a stepwise forward approach. It is possible that baseline biomarker data may be correlated leading to problems in modelling. The exploration of relationships between plasma markers described above will be used to guide selection of biomarkers as covariates.

Exploratory analyses using baseline levels of biomarkers as covariates will be performed for the primary, secondary and other efficacy analyses using a stepwise forward approach. It is possible that baseline biomarker data may be correlated leading to problems in modelling and interpretation. The exploration of relationships between plasma markers described above will be used to guide selection of covariates.

Exploratory analysis of the effect of concomitant medication usage on efficacy data will also be performed. Concomitant usage, other than statins, will be grouped by class and most common classes to be included in the exploratory analyses identified prior to unblinding the study.

11.8. Safety Analysis

Adverse event and clinical laboratory data will be reviewed on an ongoing basis during the study to evaluate the safety of the patients.

11.8.1. Extent of Exposure

A frequency distribution of the number of days of treatment completed by the patients will be produced by treatment group. Total dose received by each subject will be summarised by treatment group. Patients with treatment compliance below 80% or above 120% will be listed.

11.8.2. Adverse Events

The proportion of patients reporting each type of adverse event will be tabulated for each treatment group. Adverse events will be coded and grouped using the MedDRA dictionary. The following summaries of adverse events will be provided:

- all adverse events
- treatment-related adverse events
- adverse events leading to withdrawal from study medication
- serious adverse events.

Full details for all adverse events will be listed.

11.8.3. Clinical Laboratory Evaluations

The readings obtained at Visit 2 (randomisation) of each laboratory test will be used as the baseline value for that test. Laboratory data will be summarised for each parameter at all visits by treatment group. Absolute change from baseline will also be calculated for each subject and summarised by visit and treatment group.

A laboratory value that is on or within the reference range is considered normal. A laboratory value that is outside the testing laboratory's reference range is considered an abnormal laboratory value. A laboratory value that is above the upper limit of the reference range is considered high abnormal. A laboratory value that is below the lower limit of the reference range is considered low abnormal.

The threshold laboratory values are defined in terms of a multiplicative factor of the testing laboratory's reference range or as an absolute value. A threshold value may also be defined as a change of clinical concern from baseline. These will be defined in the Reporting and Analysis Plan (RAP). A laboratory value that is above the threshold is considered a high threshold value. A laboratory value that is below the threshold is considered a low threshold value. The proportion of patients exceeding the threshold criteria for each of these laboratory values at any post-baseline visit will be summarised by treatment group. A subject's post-baseline laboratory value will only be considered to be a threshold if it is more extreme than the baseline result and satisfies the criteria for a threshold value as defined above. A listing of patients with any threshold value during the study will also be produced.

Box plots will be used to display graphically all safety laboratory values. Any outliers will be detailed in a supporting listing.

11.8.4. Other Safety Measures

11.8.4.1. Vital Signs

For the purpose of reporting, baseline assessments will be defined as the readings obtained at Visit 2, randomisation.

Vital sign data will be summarised for each parameter at all visits by treatment group. Absolute change from baseline will also be calculated for each subject and summarised by visit and treatment group.

All vital signs data will be listed.

11.8.4.2. ECGs

ECGs will be performed at Visits 2 and 5. The results from these will be classified as normal, abnormal not clinically significant and abnormal clinically significant. These classifications will be summarised by treatment group. ECG results at Visits 2 and 5 will also be classified as no clinically significant change from baseline or clinically significant change from baseline. These classifications will also be summarised by treatment group. Clinically significant ECG findings will be listed and summarised by treatment group.

11.9. Biomarker(s) Analyses

Plaque biomarker data will be analysed as for primary variable of Lp-PLA_2 plaque activity as described in Section 11.7.1 above.

Plasma biomarker and lipid data will be analysed as for secondary variable of Lp-PLA_2 plasma activity as described in Section 11.7.2 above.

11.10. Clinical Pharmacology Data Analyses

11.10.1. Pharmacokinetic Analyses

SB-480848 plasma concentration-time data will be displayed in tables and/or graphs. SB-480848 individual plasma concentration-time data will be pooled and population pharmacokinetics will be performed using software such as NONMEM or other currently acceptable methods as permitted by the data. The influence of various covariates (e.g., age, weight, gender, race) on the pharmacokinetic parameters will be examined. Pharmacokinetic analysis will be the responsibility of the Department of Clinical Pharmacokinetics, Modeling and Simulation, Clinical Pharmacology and Discovery Medicine. All drug analysis and pharmacokinetic data will be stored in the archives of GlaxoSmithKline Research and Development.

11.10.2. Pharmacodynamic Analyses

No separate analysis of the population pharmacodynamic data will be performed but this data will be used in the pharmacokinetics/pharmacodynamics analyses, see Section 11.10.3.

11.10.3. Pharmacokinetics/Pharmacodynamics Analyses

If data permit, plasma concentrations may be subject to an exploratory analysis to correlate these data with the pharmacodynamic endpoints.

11.11. Pharmacogenetic analysis

The genotypic frequencies of each candidate gene polymorphism will be evaluated for conformity to those expected under normal conditions by employing Hardy-Weinberg Equilibrum testing. Any departure from expectation will be taken into account, possibly signalling a data error or alternatively a connection between the polymorphism and atherosclerosis. For pairs of polymorphisms located within a given gene, the degree to which alleles from the two sites are correlated (linkage disequilibrium) will also be evaluated. If the genotypes at two polymorphic sites within a gene are shown to be statistically associated with a response to investigational product, the degree of linkage disequilibium will aid interpretation in that it will indicate the extent to which the two sites are exerting independent effects. Further, a decision regarding the construction and analysis of marker haplotypes – combinations of alleles from different polymorphic sites that are inherited from one patient – will be guided by the assessment of linkage disequilibrium. For example, if there is no linkage disequilibrium between polymorphic sites, then haplotype construction will be uninformative. Differences in baseline clinical characteristics and potential contributing covariates will be summarised and compared among genotype (or haplotype) subgroups. Analyses, will be carried out to evaluate the degree of association between patient genotype (or haplotype) and selected efficacy and safety parameters. In addition to evaluating the main effects of the genotypes (or haplotypes) on the selective efficacy and safety parameters, the possibility of a treatment group by genotype (haplotype) interaction will also be explored.

Under certain circumstances, such as low recruitment of patients into the pharmacogenetic research or premature discontinuation of the clinical study, it may be decided that the samples will not be used for pharmacogenetic research and the samples will be destroyed.

11.11.1. Scope of Phamacogenetic Analysis

The analysis that may be undertaken will be limited to pharmacogenetic analyses only, based on genes, pieces of genes, or other parts of genetic material possibly associated with response to SB-480848 or its metabolites on the atherosclerotic disease process. Samples will be kept for up to 15 years after the last patient completes the study.

11.11.2. Coded sample

A coded sample will be obtained from each patient giving appropriate informed consent for pharmacogenomic analysis. A **coded sample** is one that is labelled with a patient number that can be traced or linked back to the patient only by the investigator or site staff. Samples do not carry personal identifiers (such as name).

12. STUDY ADMINISTRATION

12.1. Regulatory and Ethical Considerations

12.1.1. Regulatory Authority Approval

GSK will obtain approval to conduct the study from the appropriate regulatory agency in accordance with any applicable country-specific regulatory requirements prior to a site initiating the study in that country.

12.1.2. Ethical Conduct of the Study and Ethics Approval

This study will be conducted in accordance with "good clinical practice" (GCP) and all applicable regulatory requirements, including, where applicable, the Somerset West, Republic of South Africa, October 1996 version of the Declaration of Helsinki. Appendix 2

The investigator (or sponsor, where applicable) is responsible for ensuring that this protocol, the site's informed consent form, and any other information that will be presented to potential subjects (e.g., advertisements or information that supports or supplements the informed consent) are reviewed and approved by the appropriate IEC/IRB. The investigator agrees to allow the IEC/IRB direct access to all relevant documents. The IEC/IRB must be constituted in accordance with all applicable regulatory requirements. GSK will provide the investigator with relevant document(s)/data that are needed for IEC/IRB review and approval of the study. Before investigational product(s) and CRFs can be shipped to the site, GSK must receive copies of the IEC/IRB approval, the approved informed consent form, and any other information that the IEC/IRB has approved for presentation to potential subjects.

If the protocol, the informed consent form, or any other information that the IEC/IRB has approved for presentation to potential subjects is amended during the study, the investigator is responsible for ensuring the IEC/IRB reviews and approves, where applicable, these amended documents. The investigator must follow all applicable regulatory requirements pertaining to the use of an amended informed consent form including obtaining IEC/IRB approval of the amended form before new subjects consent to take part in the study using this version of the form. Copies of the IEC/IRB approval of the amended informed consent form/other information and the approved amended informed consent form/other information must be forwarded to GSK promptly.

12.1.3. Informed consent

Informed consent will be obtained before the subject can participate in the study. The contents and process of obtaining informed consent will be in accordance with all applicable regulatory requirements.

12.1.4. Investigator Reporting Requirements

As indicated in Section 10.10, the investigator (or sponsor, where applicable) is responsible for reporting SAEs to the IEC/IRB, in accordance with all applicable regulations. Furthermore, the investigator may be required to provide periodic safety updates on the conduct of the study at his or her site and notification of study closure to the IEC/IRB. Such periodic safety updates and notifications are the responsibility of the investigator and not of GSK.

12.2. Study Monitoring

In accordance with applicable regulations, GCP, and GSK procedures, GSK monitors will contact the site prior to the subject enrolment to review the protocol and data collection procedures with site staff. In addition, the monitor will periodically contact the site, including conducting on-site visits. The extent, nature and frequency of on-site visits will be based on such considerations as the study objective and/or endpoints, the purpose of the study, study design complexity, and enrolment rate.

During these contacts, the monitor will:

- Check the progress of the study.
- Review study data collected.
- Conduct source document verification.
- Identify any issues and address their resolution.

This will be done in order to verify that the:

- Data are authentic, accurate, and complete.
- Safety and rights of subjects are being protected.
- Study is conducted in accordance with the currently approved protocol (and any amendments), GCP, and all applicable regulatory requirements.

The investigator agrees to allow the monitor direct access to all relevant documents and to allocate his/her time and the time of his/her staff to the monitor to discuss findings and any relevant issues.

At study closure, monitors will also conduct all activities described in Section 12.4, "Study and Site Closure."

12.3. Quality Assurance

To ensure compliance with GCP and all applicable regulatory requirements, GSK may conduct a quality assurance audit. Regulatory agencies may also conduct a regulatory inspection of this study. Such audits/inspections can occur at any time during or after completion of the study. If an audit or inspection occurs, the investigator and institution agree to allow the auditor/inspector direct access to all relevant documents and to allocate

his/her time and the time of his/her staff to the auditor/inspector to discuss findings and any relevant issues.

12.4. Study and Site Closure

Upon completion of the study, the monitor will conduct the following activities in conjunction with the investigator or site staff, as appropriate:

- Return of all study data to GSK.
- Data queries.
- Accountability, reconciliation, and arrangements for unused investigational product(s).
- Review of site study records for completeness.
- Return of treatment codes to GSK.
- Shipment of PK/PD/biomarker samples to assay laboratory(ies).

In addition, GSK reserves the right to temporarily suspend or prematurely discontinue this study either at a single site or at all sites at any time for reasons including, but are not limited to, safety or ethical issues or severe non-compliance. If GSK determines such action is needed, GSK will discuss this with the Investigator (including the reasons for taking such action) at that time. When feasible, GSK will provide advance notification to the investigator of the impending action prior to it taking effect.

GSK will promptly inform all other investigators and/or institutions conducting the study if the study is suspended or terminated for safety reasons, and will also inform the regulatory authorities of the suspension or termination of the study and the reason(s) for the action. If required by applicable regulations, the investigator must inform the IEC/IRB promptly and provide the reason for the suspension or termination.

If the study is prematurely discontinued, all study data must be returned to GSK. In addition, arrangements will be made for all unused investigational product(s) in accordance with the applicable GSK procedures for the study.

Financial compensation to investigators and/or institutions will be in accordance with the agreement established between the investigator and GSK.

12.5. Records Retention

Following closure of the study, the investigator must maintain all site study records in a safe and secure location. The records must be maintained to allow easy and timely retrieval, when needed (e.g., audit or inspection), and, whenever feasible, to allow any subsequent review of data in conjunction with assessment of the facility, supporting systems, and staff. Where permitted by local laws/regulations or institutional policy, some or all of these records can be maintained in a format other than hard copy (e.g., microfiche, scanned, electronic); however, caution needs to be exercised before such action is taken. The investigator must assure that all reproductions are legible and are a

true and accurate copy of the original, and meet accessibility and retrieval standards, including re-generating a hard copy, if required. Furthermore, the investigator must ensure there is an acceptable back-up of these reproductions and that an acceptable quality control process exists for making these reproductions.

GSK will inform the investigator of the time period for retaining these records to comply with all applicable regulatory requirements. The minimum retention time will meet the strictest standard applicable to that site for the study, as dictated by any institutional requirements or local laws or regulations, or GSK standards/procedures; otherwise, the retention period will default to 15 years.

The investigator must notify GSK of any changes in the archival arrangements, including, but not limited to, the following: archival at an off-site facility, transfer of ownership of the records in the event the investigator leaves the site.

12.6. Provision of Study Results and Information to Investigators

When a clinical study report is completed, GSK will provide the major findings of the study to the investigator.

In addition, details of the study treatment assignment will be provided to the investigator to enable him/her to review the data to determine the outcome of the study for his/her subject.

12.7. Information Disclosure and Inventions

Ownership:

This includes the results of PGx assessment included in the study

All information provided by GSK and all data and information generated by the site as part of the study (other than a subject's medical records) are the sole property of GSK.

All rights, title, and interests in any inventions, know-how or other intellectual or industrial property rights which are conceived or reduced to practice by site staff during the course of or as a result of the study are the sole property of GSK, and are hereby assigned to GSK.

If a written contract for the conduct of the study which includes ownership provisions inconsistent with this statement is executed between GSK and the study site, that contract's ownership provisions shall apply rather than this statement.

Confidentiality:

All information provided by GSK and all data and information generated by the site as part of the study (other than a subject's medical records) will be kept confidential by the investigator and other site staff. This information and data will not be used by the investigator or other site personnel for any purpose other than conducting the study.

These restrictions do not apply to: (1) information which becomes publicly available through no fault of the investigator or site staff; (2) information which it is necessary to disclose in confidence to an IEC or IRB solely for the evaluation of the study; (3) information which it is necessary to disclose in order to provide appropriate medical care to a study subject; or (4) study results which may be published as described in the next paragraph. If a written contract for the conduct of the study which includes confidentiality provisions inconsistent with this statement is executed, that contract's confidentiality provisions shall apply rather than this statement.

Confidentiality of Subjects PGX Data

GSK advises that participation in this PGx research, withdrawal from this research, sample destruction, and/or PGx results should not be documented in the subject's medical records. Storage of information regarding the PGx research with source documents for the study is permissible if stored in the investigator study files.

Coded PGx samples and results will be associated with the subject's study specific number in computer databases. Coded PGx research results may be submitted to regulatory agencies as part of an investigational product submission and/or included in a research publication.

Individual genotype results will only be shared with a subject through the investigator if the subject requests to see their results and it is a requirement of a governmental agency or other legal authority that GSK make these results available. GSK will not release individual PGx results to anyone else (e.g., family members, primary care physicians, insurers, or employers) under any circumstance, unless required by law.

Publication:

For multicentre studies, the first publication or disclosure of study results shall be a complete, joint multicentre publication or disclosure coordinated by GSK. Thereafter, any secondary publications will reference the original publication(s).

Prior to submitting for publication, presentation, use for instructional purposes, or otherwise disclosing the study results generated by the site (collectively, a "Publication"), the investigator shall provide GSK with a copy of the proposed Publication and allow GSK a period of at least thirty (30) days [or, for abstracts, at least five (5) working days] to review the proposed Publication. Proposed Publications shall not include either GSK confidential information other than the study results or personal data on any subject, such as name or initials.

At GSK's request, the submission or other disclosure of a proposed Publication will be delayed a sufficient time to allow GSK to seek patent or similar protection of any inventions, know-how or other intellectual or industrial property rights disclosed in the proposed Publication.

If a written contract for the conduct of the study, which includes publication provisions inconsistent with this statement is executed, that contract's publication provisions shall apply rather than this statement.

12.8. Data Management

Subject data are collected by the investigator or designee using the Case Report Form (CRF) defined by GSK. Subject data necessary for analysis and reporting will be entered/transmitted into a validated database or data system. Clinical data management will be performed in accordance with applicable GSK standards and data cleaning procedures. Database freeze will occur when data management quality control procedures are completed. Original CRFs will be retained by GSK, while the investigator will retain a copy.

12.8.1. Pharmacogenetics

GSK may list and summarize the PGx research result from coded samples by subject number in the clinical study report. In this event, the investigator and study staff would have access to the research results and would be able to link to the results to a particular subject. The investigator and study staff would be directed to hold information confidentially.

Data from the case report forms and PGx research; using the code sample will be stored electronically. International regulations for information on computers and relevant laws on processing personal information will be followed.

13. REFERENCES

BUSS N, GREENHILL RW, ABDI MM and FIRELESS AJ. SB-480848/RSD-101JH7/1. SB-480848: 1-month oral dose toxicity study in Dogs. June 2001

CHAPMAN AND HALL SPRENT 1993, Applied Nonparametric Statistical Methods. London.P.

CHAPMAN AND HALL, J C HSU. 1996. Multiple Comparisons: Theory and Methods. London.

FINNERTY DANA, SB-480848/RSD-101LMH/1. A dose rising study to evaluate the safety, tolerability, pharmacokinetics and pharmacodynamics of single and repeat dosing of SB-480848 in healthy adults.

FINNERTY DANA, SB-480848/RSD-101PS9/1. A study to assess the pharmacokinetics, safety and tolerability of SB-480848 and atorvastatin when administered alone and in combination for 7 days to healthy adult subjects.

FREEMAN LJ, GREENHILL RW, MCMURDO AS and SMITH A, SB-480848/RSD-101JH7/1. SB480848: 1-months oral dose toxicity study in rats followed by a 1 –month off –dose period. June 2001.

MACPHEE CH, MOORES KE, BOYD HF ET AL. 1999. Lipoprotein associated phospholipase A2, platelet activating factor acetylhydrolase, generates two bioactive products during the oxidation of low density lipoprotein. Use of a novel inhibitor.. Biochem. J., 338, 479-487.

PARKARD CJ ET AL October 2000. Lipoprotein-associated phospholipase A2 as an independent predictor of coronary heart disease. New England Journal of Medicine, 343, 1148-1155.

ROSS R. April 1993. The pathogenesis of atherosclerosis: a perspective for the 1990s. Nature, 362, 801-809.

SHADDINGER, BONNIE, CHEN, SB-480848/RSD-101KB4/1. A dose-rising study to assess the preliminary safety, pharmacokinetics and pharmacodynamics of single oral doses of SB-480848 in healthy adult subjects.

TEW DG, SOUTHAN C, RICE SQJ ET AL. 1996. Purification, properties, sequencing and cloning of a lipoprotein associated serine dependent phospholipase involved in the oxidative modification of a low density lipoproteins. Arteriosclerosis, Thrombosis & Vascular Biology, 16, 591-599.

TRELFA ALICE, COMPLEY ROBERT, SB-480848/RSD-101R6S/2. A non-treatment, non-randomised study in patients scheduled for planned carotid endarterectomy, to

determine the variability of Lp-PLA2 activity in blood and atherosclerotic plaques; and evaluation of the utility of [18F] FDG-PET for imaging plaques. 03 September 2002.

14. APPENDICES

14.1. Appendix 1 Time and Events Table

	Out patient visits			In-patient Visits			Out patient visit
	Screen	Double-blind medication			Post double-blind medication		
Visit No.	1	2	3	4	5	6	7
Day (relative to randomisation)	-28 (maximum)	0 Randomisation	7 (+/-2)	14 (+/- 4)[1] Day prior to surgery	15[2] Day of surgery	16 Day after surgery	14 (+/- 3) days after last dose
Informed consent	✓						
Demography	✓						
Medical history	✓						
Chest X-ray (if one not available within previous 6 months)	✓						
Fasting blood draw for HbA1c, insulin		✓					
Blood sampling for haematology, clinical chemistry	✓	✓	✓		✓		✓
Blood sampling for peripheral lymphocytes		✓					✓
12-lead ECG		✓[5]			✓[2]		
Concomitant medication	✓	✓	✓		✓		✓
Physical examination, vital signs	✓	✓	✓		✓		✓
Baseline signs and symptoms/Adverse events	✓	✓	✓		✓		✓

Continued

Appendix 1 Time and Events Table (Continued)

	Out patient visits			In-patient Visits			Out patient visit
	Screen	Double-blind medication			Post double-blind medication		
Visit No.	1	2	3	4	5	6	7
Fasting blood draw for Lp-PLA2 and biomarkers		✓	✓3, 4	✓3,4	✓	✓4	✓
Planned carotid endarterectomy					✓		
Dispense medication		✓					
Check medication compliance			✓	✓			
PK sampling			✓3	✓3		✓	

1. Patient should be telephoned on Day 14 if not attending for visit and taking study medication for >14 days. Last dose of study medication must be taken on the day prior to surgery
2. Visit 5 procedures to be performed on the day of carotid endarterectomy (with the exception of the 12 lead ECG at Visit 5, which can be taken up to 24 hours prior to surgery (V5)
3. PK sample and PD (Lp-PLA2) at V3 can be taken at this timepoint instead of at V4 if the patient is unable to attend the Hospital early in the day of V4
4. LP-PLA2 nonfasted bloods taken at Visit 4 (3) post dose and visit 6
5. 12 lead ECG taken at V2 can be taken at V1 if it is logistically easier

14.2. Appendix 2 Declaration of Helsinki

World Medical Association

DECLARATION OF HELSINKI

Recommendations Guiding Physicians

in Biomedical Research Involving Human Subjects

Adopted by the 18th World Medical Assembly

Helsinki, Finland, June 1964

and amended by the

29th World Medical Assembly

Tokyo, Japan, October 1975

35th World Medical Assembly

Venice, Italy, October 1983

41st World Medical Assembly

Hong Kong, September 1989

and the

48th General Assembly

Somerset West, Republic of South Africa, October 1996.

INTRODUCTION

It is the mission of the physician to safeguard the health of the people. His or her knowledge and conscience are dedicated to the fulfilment of this mission.

The Declaration of Geneva of the World Medical Association binds the physician with the words, "The health of my patient will be my first consideration," and the International Code of Medical Ethics declares that, "A physician shall act only in the patient's interest when providing medical care which might have the effect of weakening the physical and mental condition of the patient."

The purpose of biomedical research involving human subjects must be to improve diagnostic, therapeutic and prophylactic procedures and the understanding of the aetiology and pathogenesis of disease.

In current medical practice most diagnostic, therapeutic or prophylactic procedures involve hazards. This applies especially to biomedical research.

Medical progress is based on research which ultimately must rest in part on experimentation involving human subjects.

In the field of biomedical research a fundamental distinction must be recognised between medical research in which the aim is essentially diagnostic or therapeutic for a patient, and medical research, the essential object of which is purely scientific and without implying direct diagnostic or therapeutic value to the person subjected to the research.

Special caution must be exercised in the conduct of research which may affect the environment, and the welfare of animals used for research must be respected.

Because it is essential that the results of laboratory experiments be applied to human beings to further scientific knowledge and to help suffering humanity, the World Medical Association has prepared the following recommendations as a guide to every physician in biomedical research involving human subjects. They should be kept under review in the future. It must be stressed that the standards as drafted are only a guide to physicians all over the world. Physicians are not relieved from criminal, civil and ethical responsibilities under the laws of their own countries.

I. BASIC PRINCIPLES

1. Biomedical research involving human subjects must conform to generally accepted scientific principles and should be based on adequately performed laboratory and animal experimentation and on a thorough knowledge of the scientific literature.

2. The design and performance of each experimental procedure involving human subjects should be clearly formulated in an experimental protocol which should be transmitted for consideration, comment and guidance to a specially appointed committee independent of the investigator and the

sponsor provided that this independent committee is in conformity with the laws and regulations of the country in which the research experiment is performed.

3. Biomedical research involving human subjects should be conducted only by scientifically qualified persons and under the supervision of a clinically competent medical person. The responsibility for the human subject must always rest with a medically qualified person and never rest on the subject of the research, even though the subject has given his or her consent.

4. Biomedical research involving human subjects cannot legitimately be carried out unless the importance of the objective is in proportion to the inherent risk to the subject.

5. Every biomedical research project involving human subjects should be preceded by careful assessment of predictable risks in comparison with foreseeable benefits to the subject or to others. Concern for the interests of the subject must always prevail over the interest of science and society.

6. The right of the research subject to safeguard his or her integrity must always be respected. Every precaution should be taken to respect the privacy of the subject and to minimise the impact of the study on the subject's physical and mental integrity and on the personality of the subject.

7. Physicians should abstain from engaging in research projects involving human subjects unless they are satisfied that the hazards involved are believed to be predictable. Physicians should cease any investigation if the hazards are found to outweigh the potential benefits.

8. In publication of the results of his or her research, the physician is obliged to preserve the accuracy of the results. Reports of experimentation not in accordance with the principles laid down in this Declaration should not be accepted for publication.

9. In any research on human beings, each potential subject must be adequately informed of the aims, methods, anticipated benefits and potential hazards of the study and the discomfort it may entail. He or she should be informed that he or she is at liberty to abstain from participation in the study and that he or she is free to withdraw his or her consent to participation at any time. The physician should then obtain the subject's freely-given informed consent, preferably in writing.

10. When obtaining informed consent for the research project the physician should be particularly cautious if the subject is in a dependent relationship to him or her or may consent under duress. In that case the informed consent should be obtained by a physician who is not engaged in the investigation and who is completely independent of this official relationship.

11. In case of legal incompetence, informed consent should be obtained from the

legal guardian in accordance with national legislation. Where physical or mental incapacity make it impossible to obtain informed consent, or when the subject is a minor, permission from the responsible relative replace that of the subject in accordance with national legislation.

Whenever the minor child is in fact able to give a consent, the minor's consent must be obtained in addition to the consent of the minor's legal guardian.

12. The research protocol should always contain a statement of the ethical considerations involved and should indicate that the principles enunciated in the present Declaration are complied with.

II. MEDICAL RESEARCH COMBINED WITH PROFESSIONAL CARE (Clinical Research)

1. In the treatment of the sick person, the physician must be free to use a new diagnostic and therapeutic measure, if in his or her judgement it offers hope of saving life, re-establishing health or alleviating suffering.

2. The potential benefits, hazards and discomfort of a new method should be weighed against the advantages of the best current diagnostic and therapeutic methods.

3. In any medical study, every patient - including those of a control group, if any - should be assured of the best proven diagnostic and therapeutic method. This does not exclude the use of inert placebo in studies where no proven diagnostic or therapeutic method exists.

4. The refusal of the patient to participate in a study must never interfere with the physician-patient relationship.

5. If the physician considers it essential not to obtain informed consent, the specific reasons for this proposal should be stated in the experimental protocol for transmission the independent committee (see I,2).

6. The physician can combine medical research with professional care, the objective being the acquisition of new medical knowledge, only to the extent that medical research is justified by its potential diagnostic or therapeutic value for the patient.

III. NON-THERAPEUTIC BIOMEDICAL RESEARCH INVOLVING HUMAN SUBJECTS (Non-Clinical Biomedical Research)

1. In the purely scientific application of medical research carried out on a human being, it is the duty of the physician to remain the protector of the life and health of that person on whom biomedical research is being carried out.

2. The subjects should be volunteers -- either healthy persons or patients for whom the experimental design is not related to the patient's illness.

3. The investigator of the investigation team should discontinue the research if in his/her or their judgement it may, if continued, be harmful to the individual.

4. In research on man, the interest of science and society should never take precedence over considerations related to the well-being of the subject.

14.3. Appendix 3 Classification of Unstable Angina

Definition of Unstable Angina

Unstable angina is defined as the presence of one or more of the following three historical features without evolution of MI by ECG or enzyme criteria (CK <3 times upper limit of normal) but with transient ST elevation or depression or T-wave inversion associated with pain, or in the absence of ECG changes the listed typical syndrome with known coronary artery disease (prior MI or documented angiographic coronary artery stenosis):

- crescendo angina (more severe, prolonged, or frequent) superimposed on a pre-existing pattern of relatively stable, exertion-related angina pectoris; or

- angina pectoris of new onset (usually within one month), which is brought on by minimal exertion; or

- angina pectoris at rest, as well as with minimal exertion.

14.4. Appendix 4 Country Specific Requirements
